# An Interferon-Related Signature in the Transcriptional Core Response of Human Macrophages to *Mycobacterium tuberculosis* Infection

**DOI:** 10.1371/journal.pone.0038367

**Published:** 2012-06-04

**Authors:** Kang Wu, Dandan Dong, Hai Fang, Florence Levillain, Wen Jin, Jian Mei, Brigitte Gicquel, Yanzhi Du, Kankan Wang, Qian Gao, Olivier Neyrolles, Ji Zhang

**Affiliations:** 1 State Key Laboratory of Medical Genomics and Sino-French Research Center for Life Sciences and Genomics, Ruijin Hospital affiliated to Shanghai Jiao Tong University School of Medicine, Shanghai, China; 2 Institute of Health Sciences, Shanghai Institutes for Biological Sciences, Chinese Academy of Sciences/Shanghai Jiao Tong University School of Medicine, Shanghai, China; 3 Key Laboratory of Medical Molecular Virology, Shanghai Medical College, Fudan University, Shanghai, China; 4 Centre National de la Recherche Scientifique, Institut de Pharmacologie et de Biologie Structurale, Toulouse, France; 5 Université de Toulouse, Université Paul Sabatier, Institut de Pharmacologie et de Biologie Structurale, Toulouse, France; 6 Department of Tuberculosis Control, Shanghai Municipal CDC, Shanghai, China; 7 Unité de Génétique Mycobactérienne, Institut Pasteur, Paris, France; Fundació Institut d’Investigació en Ciències de la Salut Germans Trias i Pujol - Universitat Autònoma de Barcelona - CIBERES, Spain

## Abstract

The W-Beijing family of *Mycobacterium tuberculosis* (*Mtb*) strains is known for its high-prevalence and -virulence, as well as for its genetic diversity, as recently reported by our laboratories and others. However, little is known about how the immune system responds to these strains. To explore this issue, here we used reverse engineering and genome-wide expression profiling of human macrophage-like THP-1 cells infected by different *Mtb* strains of the W-Beijing family, as well as by the reference laboratory strain H37Rv. Detailed data mining revealed that host cell transcriptome responses to H37Rv and to different strains of the W-Beijing family are similar and overwhelmingly induced during *Mtb* infections, collectively typifying a robust gene expression signature (“THP1r2*Mtb*-induced signature”). Analysis of the putative transcription factor binding sites in promoter regions of genes in this signature identified several key regulators, namely STATs, IRF-1, IRF-7, and Oct-1, commonly involved in interferon-related immune responses. The THP1r2*Mtb*-induced signature appeared to be highly relevant to the interferon-inducible signature recently reported in active pulmonary tuberculosis patients, as revealed by cross-signature and cross-module comparisons. Further analysis of the publicly available transcriptome data from human patients showed that the signature appears to be relevant to active pulmonary tuberculosis patients and their clinical therapy, and be tuberculosis specific. Thus, our results provide an additional layer of information at the transcriptome level on mechanisms involved in host macrophage response to *Mtb*, which may also implicate the robustness of the cellular defense system that can effectively fight against genetic heterogeneity in this pathogen.

## Introduction

*Mycobacterium tuberculosis* (*Mtb*) causes most cases of tuberculosis (TB) in humans, claiming over 1.5 million lives every year. *Mtb* isolates are more genetically diverse than previously thought, and most interestingly, such *Mtb* genetic lineages seem to be associated with bio-geographic human populations [1]–[3]. For convenience, *Mtb* lineages are thus named after their geographic origins. One group of such genetically related *Mtb* isolates was first reported from patients mainly from Beijing, China, and hence was named as “Beijing family” of *Mtb* strains [4]. At the same time period, “W” strains were described in New York City, USA [5], and exhibited relatedness with strains of the Beijing family [6]. These two strain families were then collectively named as “W-Beijing”. Since first described, the W-Beijing family has raised increasing concerns globally. Indeed W-Beijing strains of *Mtb* account for up to 50% of TB cases in East-Asia (nearly 13% in the world); moreover, several lines of evidences have pointed to their higher virulence, as compared to other strains, in various *in vitro* and *in vivo* models [4]. For instance, strains of the W-Beijing family resulted in more bacilli in the lung and earlier mortality of experimentally infected mice, as compared with strains from other families including Somali, Haarlem, Canetti, and the laboratory stain H37Rv [7], as well as in higher bacilli load in the cerebrospinal fluid and brain, and more severe clinical manifestations in rabbit model of tuberculosis meningitis, as compared to the clinical strain CDC1551 [8]. Other studies have shown that strains of the W-Beijing family induced less protective cytokines but more cell necrosis in the phorbol myristate acetate (PMA)-treated macrophage-like THP-1 cells, as compared to H37Rv [9], [10].

**Figure 1:**
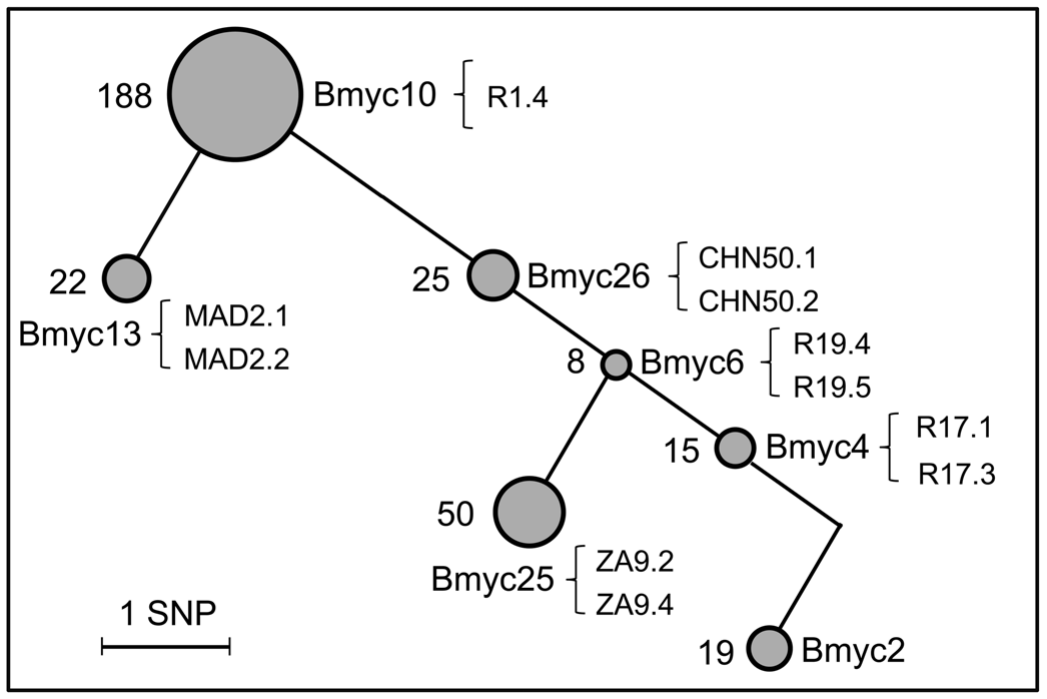
Experimental design for detecting host transcriptional responses to different *Mtb* W-Beijing strains. Genetic diversity of W-Beijing family strains, as revealed by SNPs-based genotyping (see our recent work [16] for details). In brief, 48 SNPs were characterized by sequencing 22 genes (being involved in DNA repair, replication, and recombination) in 58 W-Beijing isolates plus one non-W-Beijing isolate (Myc2). Each node represents one genotype (the same SNPs profile), with the node area proportional to the population size (the number of W-Beijing isolates in it was indicated on the left). Strains from the corresponding node used for the THP-1 infection in this study (e.g., R1.4 from Bmyc10) were indicated on the right. Lab strain H37Rv, which was also recruited for the THP-1 infection, was not shown here.

Here we aimed at exploring host macrophage response to *Mtb* W-Beijing on a genome-wide scale using gene expression profiling. Transcriptome profiling has been widely used to gain insights into host-mycobacteria interactions in various contexts [11]. In this study, we used the human macrophage-like THP-1 cell line as a model of innate immune cell because it allows to minimize the influence of host heterogeneity as compared to human blood donor-derived primary macrophages [12]. In addition to the reference laboratory strain H37Rv, a total of eleven *Mtb* strains representing six sublineages of the W-Beijing family (Figure 1) were used to infect host cells, and were subsequently profiled using whole-genome expression arrays. Through detailed data mining, we found transcriptome responses of the host were largely similar, irrespective of the *Mtb* W-Beijing subgroups tested, although it could not be excluded that there were minor differences between different strains. Accordingly, a core response gene signature was recognized (THP1r2*Mtb*-induced signature). Based on this gene signature and cross-study comparisons, we were able to identify several putative immunity-related, and more particularly interferon-related, transcription factors that might regulate the core host transcriptional response. We were also able to show the clinical relevance of such core responses with those observed in active pulmonary tuberculosis patients [13], which appears to be tuberculosis specific when compared to patients from other inflammatory diseases and pathological conditions [14], [15]. These results provide new insights into the host-pathogen cross-talk in *Mtb* infections.

## Results

### The Transcriptome Responses of THP-1 Cells to *Mtb* W-Beijing and H37Rv Strains are Broadly Similar

**Figure 2:**
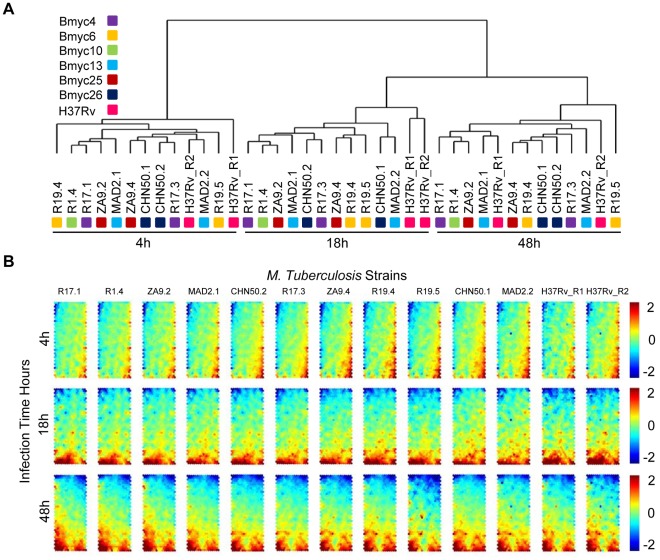
Transcriptome classification of THP-1 cells to W-Beijing strains as well as the lab strain H37Rv. (**A**) Sample classification of THP-1 transcriptome responses to *Mtb* W-Beijing and H37Rv strains.W-Beijing strains from the same node (see Figure 1) were highlighted by the same color. For example, two CHN50 strains (CHN50.1 and CHN50.2) from node Bmyc26 were colored in black. (**B**) Component plane presentation integrated self-organizing map (CPP-SOM) of host transcriptomic responses to 11 different W-Beijing family strains as well as duplicated H37Rv (columns) at 3 time points (rows). Each presentation illustrates a sample- and time-specific transcriptome map, in which all the up-regulated (represented by neurons in red), down-regulated (represented by neurons in blue) and moderately regulated (represented by neurons in yellow and green) genes were well delineated. Linking these presentations allows visual-easy comparisons of transcriptome changes across time points and strains.

In our recent work [16], we have used SNPs-based genotyping to characterize the genetic diversity of *Mtb* strains of the W-Beijing family. However, it remains unknown how host cells cope with the heterogeneity of these SNPs-genotyped strains. In an attempt to address this issue, we selected eleven W-Beijing strains from the six most representative sublineages (Figure 1), and these strains, in addition to H37Rv, were used to infect THP-1 host cells in a time-series setting. Using whole-genome expression arrays, we performed transcriptome profilings of THP-1 cells infected at three time points including early stage (4 h), intermediate stage (18 h), and late stage (48 h), as well as an uninfected sample (0 h) as a control. After array hybridization and data normalization, Absent-Present based filtering [12] was applied to select the most reliable probesets/transcripts. A total of 18,541 transcripts across the 39 infected samples (3 time points ×11 *Mtb* W-Beijing strains, 3 time points ×2 duplicated *Mtb* H37Rv strain, compared to the uninfected THP-1 cells as a control) remained and were subsequently used for unsupervised sample classifications. As shown in Figure 2A and Figure S1, samples were unambiguously classified into three groups, exactly corresponding to the infection time points, and independently of the strain genotype (*i.e.* H37Rv *vs*. W-Beijing, and among W-Beijing sublineages). When component plane presentations integrated self-organizing map (CPP-SOM) [17] were used to illustrate sample-specific transcriptome map, host cells infected by strains of different genotypes appeared to share similar global expression changes at each of all three infection time points (Figure 2B; see also pair-wise correlation coefficients in Figure S2). Moreover, the major changes were observed at the interval between 4 h and 18 h of infection, while little changes occurred thereafter. These observations suggested that host transcriptome responses do not rely on the genotype (*i.e.* sublineage) of the W-Beijing strains tested. To evaluate the possibility of whether the genetic diversity of the strains could instead be explained by function-specific differences in host cells, we also performed the sample classifications with the same parameters to Figure 2A but using genes only annotated to specific functional categories (i.e., immunity and defense from PANTHER classification system [18]). Like the results shown in Figure 2A, samples were again grouped together according to the infection duration, irrespective of the genotype of the strains (Figure S3). Further restriction using more specific immunity-related processes did not change the sample relationships (data not shown). In all, the host transcriptome responses induced by H37Rv and by different sublineages of *Mtb* W-Beijing strains are predominantly similar, and hardly broad differences as well as immune response-specific differences were detectable, even though we can not guarantee that minor differences would be observed if more biological replicates were involved. Although the small number of strains of each genotype used here may be insufficient to distinguish subtle genotype- and subgenotype-specific host cell responses, our results nevertheless suggested that a transcriptional core response of human macrophages to *Mtb* infection could be extracted from our data.

### Identification of a Common Host Transcriptome Response Signature and its Underlying Putative Transcriptional Regulators

**Figure 3:**
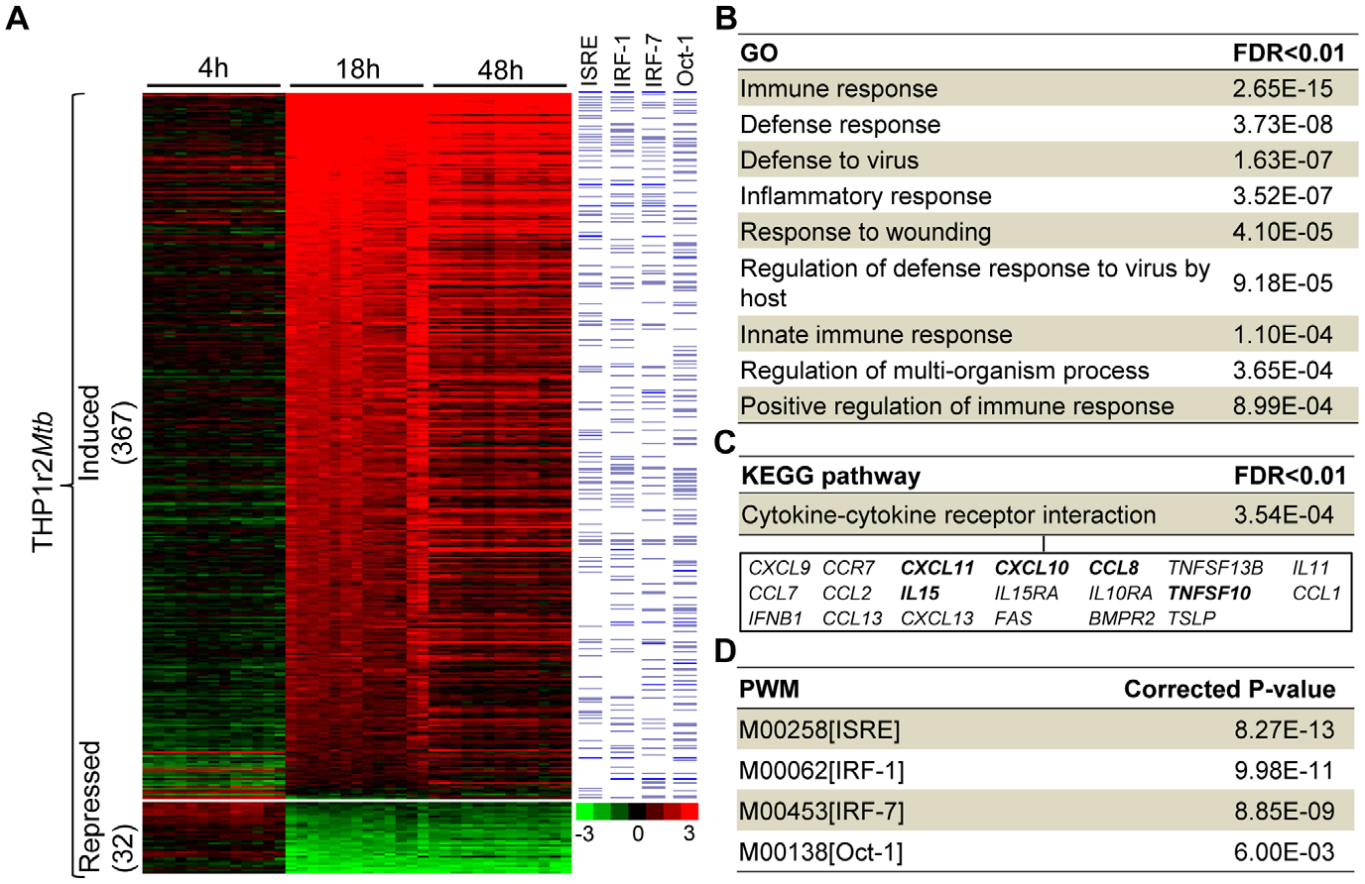
A common host transcriptome response signature and its biological characteristics. (**A**) Illustration of expression patterns of differentially expressed genes (THP1r2*Mtb*) between 4 h to 18 h after *Mtb* infection. Those genes induced are most prominent, thus representing a common host transcriptome response signature spectrum. Genes whose promoter regions (2,000 bp upstream to 200 bp downstream of transcription start site) harbouring the putative TFBSs obtained from Expander [53] were indicated by bars in blue. (**B**, **C**, and **D**) Significant functional and regulatory features in the THP1r2*Mtb*-induced signature, including GO for functional enrichments, KEGG for pathway enrichments and positional weighted matrix (PWM) for regulatory enrichments. Also listed underneath in Figure 3C are genes involved in the cytokine-cytokine receptor interaction, wherein those independently validated by qRT-PCR were highlighted in bold (see also Figure S5).

Since host transcriptome responses to *Mtb* were dramatically modulated between 4 h and 18 h, regardless of the W-Beijing sublineages (Figure 2), we next aimed to study the core responses between these two time points. It was also appealing to speculate that there might exist a common host transcriptome response signature, reflective of the robust nature of host cellular responses. To validate this speculation, we applied a LIMMA-based methodology [19] to identify genes differentially expressed between 4 h and 18 h. These genes were collectively informative as a common THP-1 response to *Mtb* (THP1r2*Mtb*; Table S2). As showed in Figure 3A, the THP1r2*Mtb* could be further divided into two parts: one for the induction at 18 h (and 48 h) as compared with 4 h (THP1r2*Mtb*-induced), the other for the repression (THP1r2*Mtb*-repressed). Interestingly, as high as 92% (367/399) of genes in the THP1r2*Mtb* are generally induced (i.e., THP1r2*Mtb*-induced). When enrichment analysis was applied to examine whether they shared functional or regulatory features [20] (see below for details), we only observed biological features associated with genes in the THP1r2*Mtb*-induced (Figures 3B–3D), and in contrast, neither functional terms nor transcription factors were significantly linked to genes in the THP1r2*Mtb*-repressed. Since genes in the THP1r2*Mtb*-repressed were trivial both in number and functional/regulatory relevance, we only considered the THP1r2*Mtb*-induced group of genes, and the latter was therefore denoted as the common host transcriptome response signature (hereinafter referred to as “THP1r2*Mtb*-induced signature”) for the subsequent analysis.

As shown in Figure 3B, functional enrichment analysis using Gene Ontology (GO) [20], [21] suggested that genes in the signature were related to cellular immune responses and defenses. Consistent with this finding, the most significant KEGG pathway [21] was only associated with cytokine-cytokine receptor interaction (Figure 3C). Most of the genes involved in the cytokine-cytokine receptor interaction have functional implications mediating host-pathogen cross-talk involving *Mtb* (Figure S4). For instance, *CCR7* [22], *CXCL13* [23], *CCL2* [24], and *IL15* [25] have been reported to be involved in the adaptive immunity to *Mtb* infection. Among potential clinical signatures reported are *CXCL9* [26], *CXCL10* [27], *CCL1* [28], and *IL11* [29], as their expression levels could differentiate some kinds of clinical manifestations such as latent TB, pulmonary, and meningeal TB. Indeed, we independently validated several randomly selected genes (i.e. *CXCL10*, *CXCL11*, *TNFSF10*, *IL15*, and *CCL8*; Figure 3C) by quantitative RT-PCR (qRT-PCR), showing that these genes were all induced in five *Mtb*-infected samples (Figure S5).

**Figure 4:**
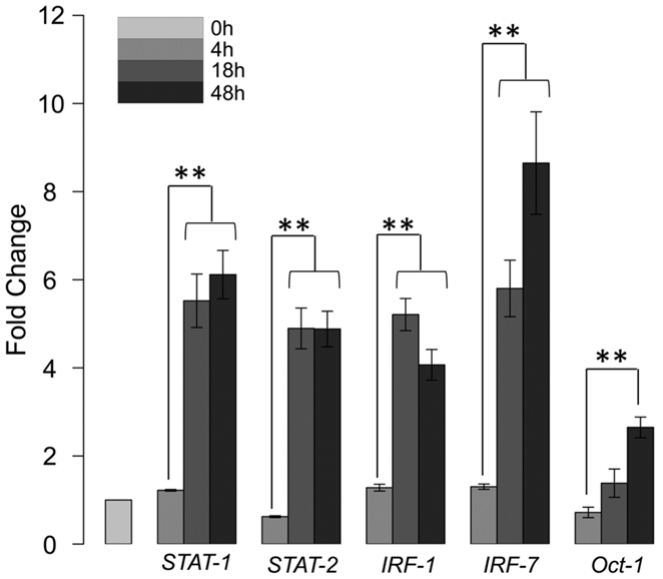
qRT-PCR validation of transcriptional regulators *STAT-1/2*, *IRF-1*, *IRF-7*, and *Oct-1*. Data were obtained from four time-series samples infected by strains of R1.4, ZA9.2, R17.1, and MAD2.1. Fold changes of the induction were illustrated and statistic tests were performed. ** for P-value<0.01.

In keeping with functional features, we also observed that the most significant TFBSs (represented in the form of PWMs) are (putative) immunity-related transcriptional regulators, including IFN-stimulated response element (ISRE), interferon regulatory factor 1 (IRF-1), IRF-7, and Oct-1 (also called POU2F1, POU class 2 homebox 1) (Figure 3D; see also the right panel of Figure 3A). Several lines of clues have pointed to their involvement in regulating the immune responses against *Mtb* infection. First, literature mining showed that these regulators had been linked to *Mtb* in several studies. Two members of the IRF family, namely IRF-1 and IRF-7, have been reported to be activated in *Mtb*-infected human monocyte-derived dendritic cells [30]. Moreover, the activation of STAT-1/2 is prerequisite for the induction of IRF-1 and IRF-7 [31]. Oct-1, a member of the POU family proteins containing six classes (POU1 to POU6), was reported to regulate type I interferon transcription [32], and was also reported to be induced in *Mtb*-infected human monocytes [33]. A recent study showed that Oct-1 may function as an anti-repressive regulator of those genes involved in immune and inflammatory responses when being exposed to oxidative stress [34]. Second, we accordingly observed the induction of *STAT-1/2*, *IRF-1*, *IRF-7*, and *Oct-1* themselves in our host cell model, which could be independently validated by qRT-PCR method (Figure 4). Then, a detailed computational survey allowed us to bridge the potential links between these multiple immune-responsive regulators (i.e., STATs, IRF-1, IRF-7, and Oct-1) and the induction of the cytokine-cytokine receptor interaction (Figure S6). By looking at the multifaceted TFBS composition in the promoter regions of the genes involved in cytokine-cytokine receptor interaction, we found that most of genes harbored at least one of the putative TFBSs of four regulators (Figure S6A). These regulators might cooperate in an additive/synergistic manner to modulate the cytokine-cytokine receptor interaction, particularly in the interferon pathways (Figure S6B).

Taken together, the above observations suggested that the genes in the THP1r2*Mtb*-induced represent a signature spectrum characteristic of core host transcriptional response to *Mtb*. The identified signature spectrum is most likely to be orchestrated by immune-responsive, interferon-related, transcription factors including STATs, IRF-1, IRF-7, and Oct-1.

### Cross-signature and Cross-module Comparisons Mirror the Transcriptional Importance of STATs, IRF-1, and IRF-7

**Figure 5:**
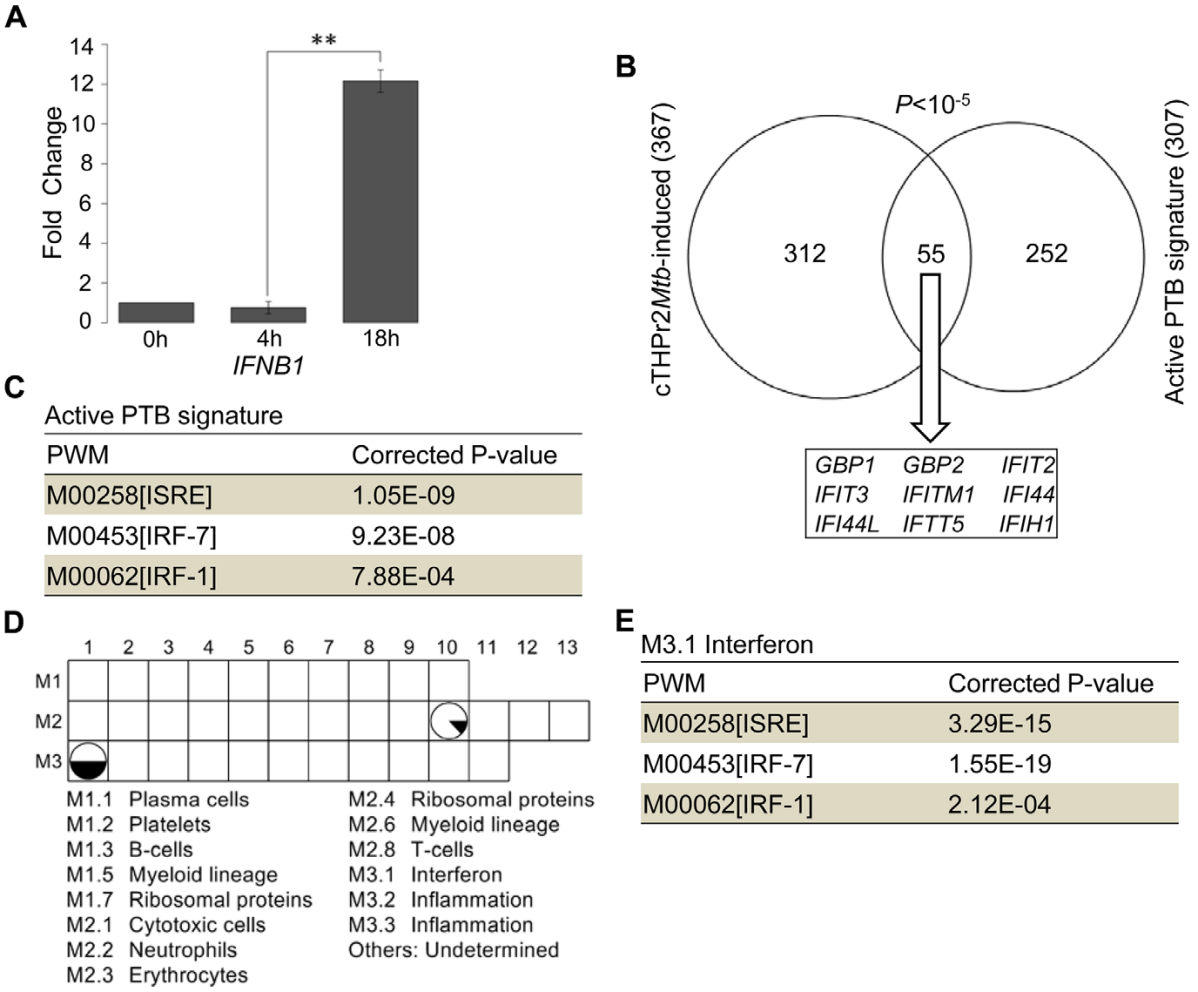
Transcriptional importance of STATs, IRF-1, and IRF-7 as supported by cross-signature and cross-module comparisons. (**A**) qRT-PCR validation of *IFNB1* induction. Data were from samples as in Figure 4. ** for P-value<0.01. (**B**) Gene overlap of the THP1r2*Mtb*-induced signature and an active pulmonary TB (PTB) signature [13]. (**C**) Regulatory/TFBS enrichment analysis in promoter regions of the active PTB signature in the form of PWM. (**D**) Gene overlap analysis of predefined modules and the THP1r2*Mtb*-induced signature. Only those modules with more than 10% of genes (represented by the proportion of black area in the pie) presented in the THP1r2*Mtb*-induced signature were displayed. Functional interpretations of modules through literature profiling [15] were indicated at lower panel. (**E**) Regulatory/TFBS enrichment analysis of module M3.1.

Binding sites enrichment of ISRE, IRF-1, and IRF-7 in the THP1r2*Mtb*-induced signature highlighted the importance of interferons in the host response to *Mtb* infection. Interferons function through their cognate receptors, and subsequently activate homo/heterodimers of STATs [35]. As validated by qRT-PCR (Figure 5A), one of the interferon-encoding genes (*IFNB1*) experienced significant activation during *Mtb* infection. Recently, one study [13] reported an interferon-inducible gene signature (393 transcripts representing 307 unique Entrez Genes) in active pulmonary TB (PTB) patients specifically. As shown in Figure 5B, we found this active PTB signature significantly overlapped with our THP1r2*Mtb*-induced signature (55 unique Entrez Genes; *P*<10^−5^, Fisher’s exact test). Interferon-inducible genes, such as *GBP1*, *GBP2*, *IFIT2*, *IFIT3*, *IFITM1*, *IFI44*, *IFI44L*, *IFIT5*, and *IFIH1* [13], [36], were largely represented in overlapping genes. In addition, regulatory enrichment analysis showed that ISRE (corrected P-value: 1.05×10^−9^), IRF-7 (corrected P-value: 9.23×10^−8^), and IRF-1 (corrected P-value: 7.88×10^−4^) were also overrepresented in this active PTB-correlated whole-blood transcript signature (Figure 5C). To further explore the relatedness of our identified THP1r2*Mtb*-induced signature to the interferon response in an unbiased manner, we chose a series of gene modules that were constructed by a modular-based data-mining method [15]. Genes in each of these modules were coordinately expressed across one or more diseases and were associated with certain functional characteristics. Through comparison, we found that two modules, M2.10 and M3.1, largely overlapped with our THP1r2*Mtb*-induced signature (Figure 5D). Although no general functions in module M2.10 have been assigned yet, literature profiling [37] had shown that those genes in M2.10 are as expected in the case of myeloid cells, such as THP-1 cells, infected with an inflammatory pathogen, such as *Mtb*. Module M3.1 is an interferon-relevant module, in which approximately half of the genes can be found in the THP1r2*Mtb*-induced signature. Regulatory enrichment analysis of the Module M3.1 also suggested its potential regulation by ISRE (corrected P-value: 3.29×10^−15^), IRF-7 (corrected P-value: 1.55×10^−19^), and IRF-1 (corrected P-value: 2.12×10^−4^) (Figure 5E). These comparative analyses provide another layer of support for the regulatory importance of STATs, IRF-1, and IRF-7 in the host response to *Mtb* infection.

### The THP1r2*Mtb*-induced Signature can be Used as a General Indicator Distinguishing Human Tuberculosis Patients of Different Disease Degrees and of Different Therapy Periods, and Appears to be Tuberculosis Specific

Since the THP1r2*Mtb*-induced signature reflects common immune responses to *Mtb* infection, both in terms of functional and regulatory relevance, we therefore further explored the possibility of using this signature to monitor the human tuberculosis patients with different extent of the disease. To do so, we viewed the signature as a gene set for gene set enrichment analysis (GSEA) [38] using the blood transcriptome data from human tuberculosis patients [13] (see Materials and Methods). As shown in Table 1 (also see Figure S7), the THP1r2*Mtb*-induced signature was significantly and positively correlated with the active pulmonary TB (PTB) as compared to latent TB (LTB) and healthy controls (Con), using any of the three datasets including London patient-based training set, London patient-based test set and South Africa patient-based validation set. When the separate cell sets were used in which bloods in PTB were separated into neutrophils, monocytes, CD4^+^ and CD8^+^ T cells, significant positive correlations were also observed (Table 1 and Figure S8), with the most positive for neutrophils (Neut_PTB *vs.* Neut_Con with NES = 3.27 and FDR = 0). This is consistent with the fact that neutrophils account for the largest white cell population in blood compared to the other three (i.e., monocytes, CD4^+^ T cells, CD8^+^ T cells) [13]. In addition to the correlation with the human tuberculosis patients, we found that the THP1r2*Mtb*-induced signature was also relevant to the PTB therapy as well (Table 1 and Figure S9). GSEA analyses of the signature using longitudinal study showed that, compared to the healthy control, the positive correlation decreased gradually as the therapy was prolonged from the pre-therapy (PTB_0m *vs.* Con with NES = 2.95 and FDR = 0) to 2-month therapy (PTB_2m *vs.* Con with NES = 2.62 and FDR = 0) and to 12 months after the therapy (PTB_12m *vs.* Con with NES = 1.43 and FDR = 0). Although the significant positive correlations were still observed for both PTB_2m and PTB_12m against the healthy control, significant negative correlations were instead observed when the comparisons were made against the pre-therapy PTB (PTB_2m *vs.* PTB_0m with NES = −2.08 and FDR = 0; PTB_12m *vs.* PTB_0m with NES = −2.96 and FDR = 0). These results clearly showcase the discriminative power of the THP1r2*Mtb*-induced signature in distinguishing human tuberculosis patients of different disease degrees and of different therapy periods.

**Table 1:** GSEA of the THP1r2*Mtb*-induced signature using transcriptome data from human tuberculosis patients.

Cohort^1^	Groupcomparison^2^	NES^3^	FDR^4^	Correlation^4^
Training set	PTB *vs*. Con	3.23	0.000	Positive
	PTB *vs*. LTB	3.00	0.000	Positive
	LTB *vs*. Con	−1.22	0.071	Null
Test set	PTB *vs*. Con	3.46	0.000	Positive
	PTB *vs*. LTB	3.12	0.000	Positive
	LTB *vs*. Con	2.37	0.000	Positive
Validation set	PTB *vs*. LTB	3.27	0.000	Positive
Test set_seperated	Neut_PTB *vs.*Neut_Con	3.27	0.000	Positive
	Mono_PTB *vs*.Mono_Con	2.98	0.000	Positive
	CD4_PTB *vs*.CD4_Con	1.86	0.000	Positive
	CD8_PTB *vs*.CD8_Con	1.70	0.000	Positive
Longitudinal study	PTB_0m *vs*. Con	2.95	0.000	Positive
	PTB_2m *vs*. Con	2.62	0.000	Positive
	PTB_12m *vs*. Con	1.43	0.000	Positive
	PTB_12m *vs*.PTB_0m	−2.96	0.000	Negative
	PTB_2m *vs*.PTB_0m	−2.08	0.000	Negative

1 Traning set: all the donors, which were defined as PTB, LTB or healthy, were recruited from London, UK. Test set: all the donors were from London UK. Validation set: all the donors were from Cape Town, South Africa. Test set_seperated: purified neutrophils, monocytes, CD4^+^ T cells, and CD8^+^ T cells from both PTB and healthy controls. Longitudinal study: PTB patients before drug treatment, 2 months after drug treatment, and 12 months after drug treatment.

2 Group-group contrasted and ranked by LIMMA-based method.

3 (+) NES for positive correlation, (−) NES for negative correlation.

4 Significance of correlation, an FDR of 0.05 or lower was accepted as statistically significant for NES (“Positive” or “Negative”), otherwise “Null”. See Figures S7,S8,S9 for plot graph of each group comparison. See Figure S10 for CPP-SOM graph of expression data of each donor.

For the clinical use of our identified signature, it also requires extensive investigations and detailed evaluations of whether this signature is *Mtb* specific. For such a purpose, we selected two transcriptome datasets of peripheral-blood mononuclear cells, which were isolated from human patients with other inflammatory diseases and pathological conditions [14], [15], and used them for the GSEA analysis of the THP1r2*Mtb*-induced signature. As showed in Table 2, no correlation was observed between this signature and any of acute infections caused by *Streptococcus pneumoniae* (*Strep*), *Staphylococcus aureus* (*Staph*), Influenza A, and *E. coli* [14] (see also Figure S11). Similarly, we also observed no significant correlation of the signature with any inflammatory diseases and pathological conditions reported in [15] (Table 3 and Figure S13), including type I diabetes, *Staph* infection, liver transplant undergoing immunosuppressive therapy, *E. coli* infection, metastatic melanoma, systemic lupus erythematosus (SLE), and systemic juvenile idiopathic arthritis (JIA). These observations suggest that the THP1r2*Mtb*-induced signature might be specific to *Mtb*. This preliminary analysis also indicates the worth of future studies in further clarifying the *Mtb*-specific nature of our identified signature (and its derivatives) to justify its potential value in the clinical settings.

**Table 2:** GSEA of the THP1r2*Mtb*-induced signature using transcriptome data from human patients with acute infections.

Group comparison^1^	NES^2^	FDR^3^	Correlation^3^
*Strep vs.* Con	−0.58	1.00	Null
*Staph vs.* Con	−0.77	0.87	Null
Influenza A *vs.* Con	−0.88	0.69	Null
*E. coli vs.* Con	−0.65	0.96	Null

1 *Strep*: *Streptococcus pneumonia* infection, *Staph*: *Staphylococcus aureus* infection.

2,3 The same as in Table 1.

**Table 3:** GSEA of the THP1r2*Mtb*-induced signature using transcriptomes from patients with other inflammatory or pathological conditions.

Group comparison^1^	NES^2^	FDR^3^	Correlation^3^
Type I diabetes *vs.* Con	−1.34	0.112	Null
*Staph vs.* Con	−1.09	0.305	Null
Liver-transplant *vs.* Con	−0.79	0.892	Null
*E. coli vs.* Con	−0.91	0.646	Null
Melanoma *vs.* Con	−0.91	0.641	Null
SLE *vs.* Con	−1.14	0.396	Null
JIA *vs.* Con	−0.74	1.000	Null

1 *Staph*: *Staphylococcus aureus* infection, Liver-transplant: liver-transplant undergoing immunosuppressive therapy, Melanoma: metastatic melanoma, SLE: systemic lupus erythematosus, JIA: systemic juvenile idiopathic arthritis.

2,3 The same as in Table 1.

## Discussion

### The Robust Nature of Immune Cells in Responding to *Mtb*

How immune cells cope with the heterogeneous nature of a pathogen is key to understand host-pathogen cross-talk. Using THP-1 cells as a host immune cell system, we have profiled time-course host transcriptome responses to distinct *Mtb* strains of the W-Beijing family. Although these strains show genetic diversity (Figure 1), surprisingly these differences were largely ignored by immune cells in terms of transcriptional responses (Figure 2). To exclude the possibility that the effects of genetic diversity on the host were caused by functional biases, we have restricted our analysis based on specific functional categories and found the sample relationships were robustly preserved (Figure S3). More importantly, this genome-wide expression resource provides useful clues for understanding the ability of host cells to respond to this genetically diversified pathogen. From the temporal point of view, THP-1 cells after 4 h of infection did not exhibit obvious changes at the transcriptional level. From 4 h to 18 h, dramatic transcriptional changes occurred. Thereafter, this state of transcriptome lasted to 48 h (Figure 2), after which host cells underwent extensive death. With contrast to transcriptomes at 4 h (the early phase of THP-1 response), the transcriptomes at 18 h and 48 h represent balanced states between THP-1 cells and *Mtb* strains. From the epidemiological point of view, different groups of strains within W-Beijing family are known to have different transmissibility [16], [39], [40]. This different transmissibility may rely on their differential ability to modify the host immune response (both innate and adaptive immunity). However, we have shown that host transcriptional responses, at least in THP-1 cells, are not sensitive to the strain-to-strain variability. This is consistent with our recent report showing that cytokine secretion by human primary macrophages and dendritic cells was unaffected by the genetic diversity within the W-Beijing family [41]. The temporal duration of infections dwarfs genetic diversity of strains, which highly suggests the robustness of host cells to invasions from this evolving pathogen.

### The Potential Regulatory Mechanisms Underlying the Robustness of Host Cell Responses to *Mtb*

Time-specific and genotype-irrelevant host transcriptome responses have allowed us to identify a common signature of host responses during the shift from 4 h to 18 h (Figure 3). Using the concept of reverse engineering, functional and regulatory features have been inferred from this signature. We have observed that most of the genes in this signature are of functional relevance to immune-related processes and pathways, indicating rigorous activation of the immune response. It has been known for over a decade that immune cells, in particular phagocytes such as macrophages and dendritic cells, exhibit a core gene expression profile when exposed to various microorganisms, even from diverse phyla [42], [43]. The core of immune-related genes found here in *Mtb*-infected THP-1 cells is consistent with previous reports in other phagocytes [12], [14], [44], [45]. Based on computationally predicted TFBSs, we have also identified several transcription factors (i.e., STATs, IRF-1, IRF-7, and Oct-1); the induction of both their putative targeted genes and their own may be largely responsible for the observed common host responses (Figures 3 and 4). Interestingly, transcriptional regulators such as NF-κB [46], which are commonly believed to be pivotal in the immune response against pathogen, were not over-represented in genes significantly regulated at 18 h compared to 4 h. This is likely because the NF-κB (and genes influenced by this master regulator) might be involved before 18 h and thus we were not able to capture such involvement. Alternatively, transcriptional regulators identified in this study might compensate the absence of NF-κB. In the future, follow-up studies may be needed to clarify its exact regulatory roles.

The potential involvement of the immune-relevant transcriptional regulators in common host transcriptional responses has several important implications. First, STATs, IRF-1, IRF-7, and Oct-1 highlight the importance of interferons in host response to *Mtb*-infection. Indeed, one of the interferon-encoding genes (*IFNB1*) displays significant activation in our data (Figure 5A). Further comparison with a clinical patient-linked signature and modules provides additional support for the importance of interferon-inducible genes and their putative regulators STATs, IRF-1, and IRF-7 (Figures 5B–5E). Second, enrichment of Oct-1 binding site also implies its regulatory importance in the host response to *Mtb* infection. Probably, Oct-1 cooperates with other regulators to form a regulatory network. In addition to our host model, two *in vitro* studies have previously reported the simultaneous activation of STATs, IRF-1, IRF-7, and Oct-1 after infection of human monocytes or dendritic cells by *Mtb* strains [31], [33]. Third, our preliminary analysis suggests the mechanistic links between immune-relevant transcriptional regulators and the cytokine-cytokine receptor interaction (Figure S6). We suspect that the modulation of genes involved in the cytokine-cytokine receptor interaction by these regulators, probably in an additive/synergistic manner, may underpin the robustness of host cell responses to *Mtb* W-Beijing strains.

### Perspectives on Future Studies of Host Transcriptional Responses to *Mtb* Pathogens

In additional to W-Beijing family strains, we have also profiled transcriptomic responses of THP-1 cells to the laboratory strain H37Rv. We observed no obvious host transcriptome differences between cells infected with W-Beijing strains and with H37Rv. No matter their origins, samples are generally grouped together according to the infection duration (Figures 2A, S1, and S3). This observation needs further clarification involving more samples in future studies. Since this study has revealed the commonality of the host cell response to infection by different W-Beijing strains, it is also needed to investigate whether the host immune system responds in a similar or different way, upon infections of different strains from non-W-Beijing families.

We expect that, in the near future, the TB community could benefit from the use of the signature identified in this study. Although all of genes in this signature are potentially useful, practically only some of them should be taken as a priority. Based on their expertise and experimental convenience, researchers might only show interests in some genes for detailed functional studies. In particular, those genes involved in cytokine-cytokine interactions should be the focus of future studies for their clinical value as prognosis and diagnosis. Although currently we are tempted to these small-scale studies, we are always encouraged by signature-based microarray analysis of human TB patients once the techniques allow us to do so, both scientifically reliable and financially feasible.

In this setting, we have also shown that this *in vitro* THP-1 host cell model has great relevance to clinical tuberculosis. Processing transcriptome data from human tuberculosis patients, we have illustrated that the identified common host response signature is positively correlated with the PTB patients, no matter whether the patients were recruited from intermediate (UK) or high-burden (Sough Africa) regions (Table 1) [13], and accordingly is also correlated with patients of different therapy periods. Furthermore, we illustrated that the THP1r2*Mtb*-induced signature is tuberculosis specific and is irrelevant to human patients with other inflammatory diseases and pathological conditions (Tables 2 and 3). For these reasons and because of this signature potentially regulated by STATs, IRF-1, IRF-7, and Oct-1, we anticipate that establishing genome-wide links among these predicted regulators through high-throughput approaches (e.g. ChIP-seq [47]) might not only extend the knowledge we obtained from this study, but also allow us to explore their clinical value in the future.

## Materials and Methods

### *Mtb* Strains, Cell Line and Infection

Strains were grown at 37°C in Middlebrook 7H9 broth (BD Difco) supplemented with 10% albumin-dextrose-catalase (ADC), 0.5% glycerol and 0.05% tween-80. THP-1 cell line, which was obtained from cell bank of Chinese Academy of Sciences (Shanghai, China), was maintained at 37°C in 5% CO_2_ in RPMI 1640 (Gibco) supplemented with 10% FBS. Prior to infection, THP-1 cells were induced to differentiate into macrophages with 200 nM PMA (Sigma) for 24 h. Then, THP-1 cells were infected at multiplicities of infection (MOI) of 5. After 4, 18 and 48 h, the infected cells were collected for each of 11 *Mtb* W-Beijing strains. Also, THP-1 cells were infected by the reference laboratory strain H37Rv, and the infected cells at 4, 18, and 48 h were collected (in duplicate). A total of 40 samples (including the 33 W-Beijing-infected samples, the 6 H37Rv-infected samples, and an uninfected sample at 0 h as a control) were subjected to the RNA extraction for microarray hybridization.

### Microarray Hybridization and Data Mining

RNA was treated for hybridization with HG-U133 Plus 2.0 Array according to the Affymetrix protocol. Then, arrays were scanned by a high-resolution scanner and the scanned images were converted to cell intensity file (.CEL files) using GeneChip Operating Software (GCOS). CEL raw expression data were normalized and filtered as previously described [12]. Briefly, these CEL raw expression data were first normalized using Robust Multi-array Averaging (RMA) with quantile normalization in R (Bioconductor) [19]. Detection call-based filter was then applied to remove all the probesets whose expression values were consistently below an empirically determined value of minimum sensitivity. This value was calculated according to the 95th percentile of all the ‘Absent’ call-flagged signals of the entire dataset. Any expression values below this value are considered as being technically unreliable. Since the uninfected sample was served as a common control for the comparison, probesets with expression values less than this empirical value at the uninfected control were also removed. Finally, expression values of probesets in 39 infected samples were subtracted by their cognate values in the uninfected control (0 h).

After the normalization and pre-filtering above, a gene expression matrix (see Table S1) was constructed, containing 18,541 probesets (representing 10,315 unique Entrez Genes) across 39 samples, that is, time-courses (4 h, 18 h, and 48 h) of THP-1 cells infected by each of 11 *Mtb* W-Beijing strains as well as H37Rv (in duplicate). This matrix was first subjected to Cluster3.0 [48]/TreeView-1.0.8 [49] with Euclidean distance for unsupervised sample classification. The pair-wise Pearson’s correlation coefficients were also calculated to show a high degree of similarity for different strains within the same time point. For gene clustering and visualization, the gene expression matrix was also subjected to component plane presentation integrated self-organizing map (CPP-SOM) [17], a component of topology-preserving selection and clustering (TPSC) package [50]. Specifically, the input data were first trained using the SOM algorithm with the Epanechikov neighborhood kernel. The trained map was then visualized by CPP to display sample-specific transcriptome changes.

Linear Models for Microarray Data (LIMMA) [19], [51] was applied to identify those differentially expressed genes between any two successive time points. LIMMA used linear models and empirical Bayes methods (the moderated t-statistic) in assessing differential expression. The criteria for identifying the top significant probesets for the designed contrast was based on adjusted P-value<0.01, as corrected using Benjamini and Hochberg procedure [19], [51]. Under such criteria, all genes identified as being differentially expressed from 4 h to 18 h were expressed by 2-folds or more. These highly modulated genes were further divided into two groups: the induced group (THP1r2*Mtb*-induced) and the other repressed (THP1r2*Mtb*-repressed). The former overwhelmingly dominates the latter; those genes in the THP1r2*Mtb*-induced group collectively typify a molecular signature of common host transcriptome responses to *Mtb*. Notably, genes in this signature are not only of statistical significance (adjusted P-value<0.01), but also of biological significance (at least 2-fold increase from 4 h to 18 h).

### Functional and Regulatory Enrichment Analysis

Functional enrichment analysis using Gene Ontology (GO) and KEGG Pathways was conducted to interpret the gene set of interest (e.g., those genes in THP1r2*Mtb*-induced signature). This analysis was implemented by Database for Annotation, Visualization and Integrated Discovery (DAVID) v6.7 for identifying enriched GO and KEGG pathway, based on Benjamini and Hochberg-derived FDR (<0.01) [21], [52]. For regulatory enrichment analysis, PRomoter Integration in Microarray Analysis (PRIMA) was used to identify putative transcriptional regulators for a given gene set compared to background (entire Entrez Genes) (Bonferroni-corrected P-value<0.01) [53]. Based on the known transcription factor binding sites (as represented by positional weight matrix, PWM), PRIMA scanned putative binding sites of promoter sequences spanning promoter regions from 2,000 bp upstream to 200 bp downstream of the transcription start site.

### Gene Set Enrichment Analysis (GSEA) of the THP1r2*Mtb*-induced Signature against Transcriptome Data from Human Tuberculosis Patients, and from Human Patients with other Inflammatory Diseases and Pathological Conditions

GSEA [38] was applied to determine whether genes in a gene set (i.e., the THP1r2*Mtb*-induced signature) show statistically significant differences between two biological states (such as tuberculosis patients *vs.* the healthy controls). Specially, it aimed to determine the extent to which genes in THP1r2*Mtb*-induced signature as a whole were overrepresented at the top or bottom of a predefined list of ranked genes (derived from publicly available transcriptome datasets). Three transcriptome datasets were retrieved from NCBI GEO with accession number GSE19491 [13], GSE6269 [14] and GSE11907 [15] for the GSEA analysis.

In GSE19491, it contains transcriptomes from 498 whole blood samples that can be subdivided into several cohort sets, including training set, test set, validation set, longitudinal set, separated cell set, and others. Here, it was used to test the extent to which the THP1r2*Mtb*-induced signature was relevant to active pulmonary tuberculosis patients and their clinical therapy. As compared to the corresponding controls, these cohort sets were illustrated by CPP-SOM into a single visualization (see Figure S10), displaying: 1) London patients with the active pulmonary TB (PTB) and latent TB (LTB) for training set, 2) London patients with PTB and LTB for test set, 3) South Africa PTB for validation set, 4) bloods in PTB being separated into neutrophils (Neut_PTB), monocytes (Mono_PTB), CD4^+^ (CD4_PTB) and CD8^+^ (CD8_PTB) T cells for separated cell set, 5) PTB at pre-treatment (PTB_0m), 2 months after treatment initiation (PTB_2m), and 12 months after treatment initiation (PTB_12m).

In additional, another two datasets (GSE6269 and GSE11907), which were generated by Affymetrix HG-U133A Array platform, were also used to test whether the THP1r2*Mtb*-induced signature was relevant to human patients with other inflammatory diseases and pathological conditions. In GSE6269, it contains transcriptomes from 97 peripheral-blood mononuclear cell (PBMC) samples, of which the pediatric donors had the following conditions/acute infections: 1) *E. coli* infection, 2) Influenza A infection, 3) *Staphylococcus aureus* (*Staph*) infection, and 4) *Streptococcus pneumonia* (*Strep*) infection. The donor-specific profiles, as compared to the healthy donors, were visualized by CPP-SOM (see Figure S12). In GSE11907, it contains transcriptomes from 304 PBMC samples, of which the donors belonged to one of the following groups: 1) *E. coli* infection, 2) Systemic juvenile idiopathic arthritis (JIA), 3) Systemic lupus erythematosus (SLE), 4) Liver-transplant recipients undergoing immunosuppressive therapy, 5) Metastatic melanoma, 6) Type I diabetes, 7) *Staphylococcus aureus* (*Staph*) infection. Similarly, the healthy control-subtracted, donor-specific profiles were visualized in Figure S14.

For each group comparison above, a list of ranked genes was predefined by LIMMA supervised analysis, followed by GSEA of THP1r2*Mtb*-induced signature. GSEA reported a normalized enrichment score (NES) and a false discovery rate (FDR) for interpreting the results. NES reflects the enrichment pattern of a gene set in a ranked list of genes; positive NES means its enrichment at the top, and negative one at the bottom. The significance of NES can be accessed by the FDR. GSEA results (i.e., NES and FDR) were detailed in Figures S7,S8,S9, S11, and S13. An FDR of 0.05 or lower was accepted as indicating statistical significance for NES (positive or negative) as a whole. Notably, this significance is not specific to individual genes but rather in terms of the whole signature. The full explanations underlying the GSEA algorithm can be found in the original paper [38].

### Quantitative RT-PCR (qRT-PCR) Analysis

qRT-PCR was performed with SYBR Green PCR Master Mix (TOYOBO) in an ABI 7900HT Sequence Detection System and analyzed with SDS2.3 Software (Applied Biosystems). 500 ng of total RNA was reversely transcribed with SuperScript II Reverse Transcriptase (Invitrogen). Genes for qRT-PCR validation include *CXCL10*, *CXCL11*, *TNFSF10*, *IL15*, *CCL8*, *STAT-1*, *STAT-2*, *IRF-1*, *IRF-7*, *and Oct-1* with *GAPDH* as internal control. Primers were listed in Table S3.

### Accession Number

The transcriptome profilings of THP-1 cells infected by 11 W-Beijing strains and H37Rv (duplicated) have been deposited in NCBI GEO under accession number GSE29628.

## Supporting Information

Figure S1 **Principal component analysis of transcriptome of THP-1 cells infected by** ***Mtb*** **strains.** Transcriptome profiles of THP-1 cells infected by W-Beijing strains as well as the lab strain H37Rv at different time points were colored as indicated. The number of those genes significantly upregulated and downregulated between any adjacent time points were colored red and green respectively, as identified using LIMMA.
(TIF)

Figure S2 **The visualization of pair-wise correlations between samples.** The colors relate to Pearson’s correlation coefficient values, with deeper red colors indicating higher correlations.
(TIF)

Figure S3 **Sample classification based on genes annotated to immunity and defense functional categories.** Immunity and defense related genes (1,091 probesets) was used for unsupervised sample classification. Samples infected by strains from the same node were indexed with the same color as in Figure 2A.
(TIF)

Figure S4 **The functional implications of cytokine-cytokine receptor interactions in mediating host-** ***Mtb*** **cross-talk.** Expression pattern (left panel) of genes involved in cytokine-cytokine receptor interaction and their functional implications in TB process (right panel).
(TIF)

Figure S5 **qRT-PCR validation of** ***CXCL10*** **(A),** ***CXCL11*** **(B),** ***TNFSF10*** **(C),** ***IL15*** **(D), and** ***CCL8*** **(E).** Expression of five genes were validated in five time-series samples infected by strains of R1.4, ZA9.2, R17.1, MAD2.1, and H37Rv. The fold changes at 4 h and 18 h of infection relative to 0 h (prior to infection) were log_2_-transformed.
(TIF)

Figure S6 **Potential involvement of STATs, IRF-1, IRF-7, and Oct-1 in cooperatively regulating cytokine-cytokine receptor interactions. (A)** Relative locations of putative binding sites (ISRE, IRF-1, IRF-7, and Oct-1) in promoter regions of genes involved in cytokine-cytokine receptor interactions. **(B)** Putative target overlaps among STATs, IRF-1, IRF-7, and Oct-1 on genes in pathway of cytokine-cytokine receptor interactions.
(TIF)

Figure S7 **GSEA of the THP1r2** ***Mtb*** **-induced signature using transcriptome data from tuberculosis patients with different disease degrees.**
(TIF)

Figure S8 **GSEA of the THP1r2** ***Mtb*** **-induced signature using transcriptome data from different cell populations in PTB patients.**
(TIF)

Figure S9 **GSEA of the THP1r2** ***Mtb*** **-induced signature using transcriptome data from tuberculosis patients with different therapy periods.**
(TIF)

Figure S10 **CPP-SOM of transcriptome data from human tuberculosis patients.** The Series Matrix File, which contains expression data of 498 samples, was downloaded from NCBI GEO (accession number: GSE19491). The expression data from PTB, PTB with different therapy periods, LTB, or separated leucocyte populations of PTB were subtracted by their cognate healthy controls. Then, the subtracted profiles were visualized by CPP-SOM as in Figure 2B.
(TIF)

Figure S11 **GSEA of the THP1r2** ***Mtb*** **-induced signature using data from human patients with other acute infections.**
(TIF)

Figure S12 **CPP-SOM of transcriptome data from human patients with other acute infections.** The raw data were downloaded from NCBI GEO (accession number: GSE6269). The data were normalized and filtered in the same way as our THP-1 transcriptome data. The acute infection data were subtracted by the healthy controls, and visualized by CPP-SOM as in Figure 2B.
(TIF)

Figure S13 **GSEA of the THP1r2** ***Mtb*** **-induced signature using transcriptomes from patients with other inflammatory or pathological conditions.**
(TIF)

Figure S14 **CPP-SOM of transcriptome data from patients with other inflammatory or pathological conditions.** The raw data were downloaded from NCBI GEO (accession number: GSE11907). The data were normalized, filtered using the same as our THP-1 transcriptome data.
(TIF)

Table S1 **TB expression matrix (18541×39).**
(TXT)

Table S2 **THP1r2** ***Mtb*** **, and biological characteristics in THP1r2** ***Mtb*** **-induced signature.**
(XLS)

Table S3 **Primers for real-time quantitative RT-PCR.**
(XLS)
