# Supplementary figures and images for: An Interferon-Related Signature in the Transcriptional Core Response of Human Macrophages to Mycobacterium tuberculosis Infection
Source: PLoS One. 2012 Jun 4;7(6):e38367. doi: 10.1371/journal.pone.0038367 (PMC3366933; doi:10.1371/journal.pone.0038367)

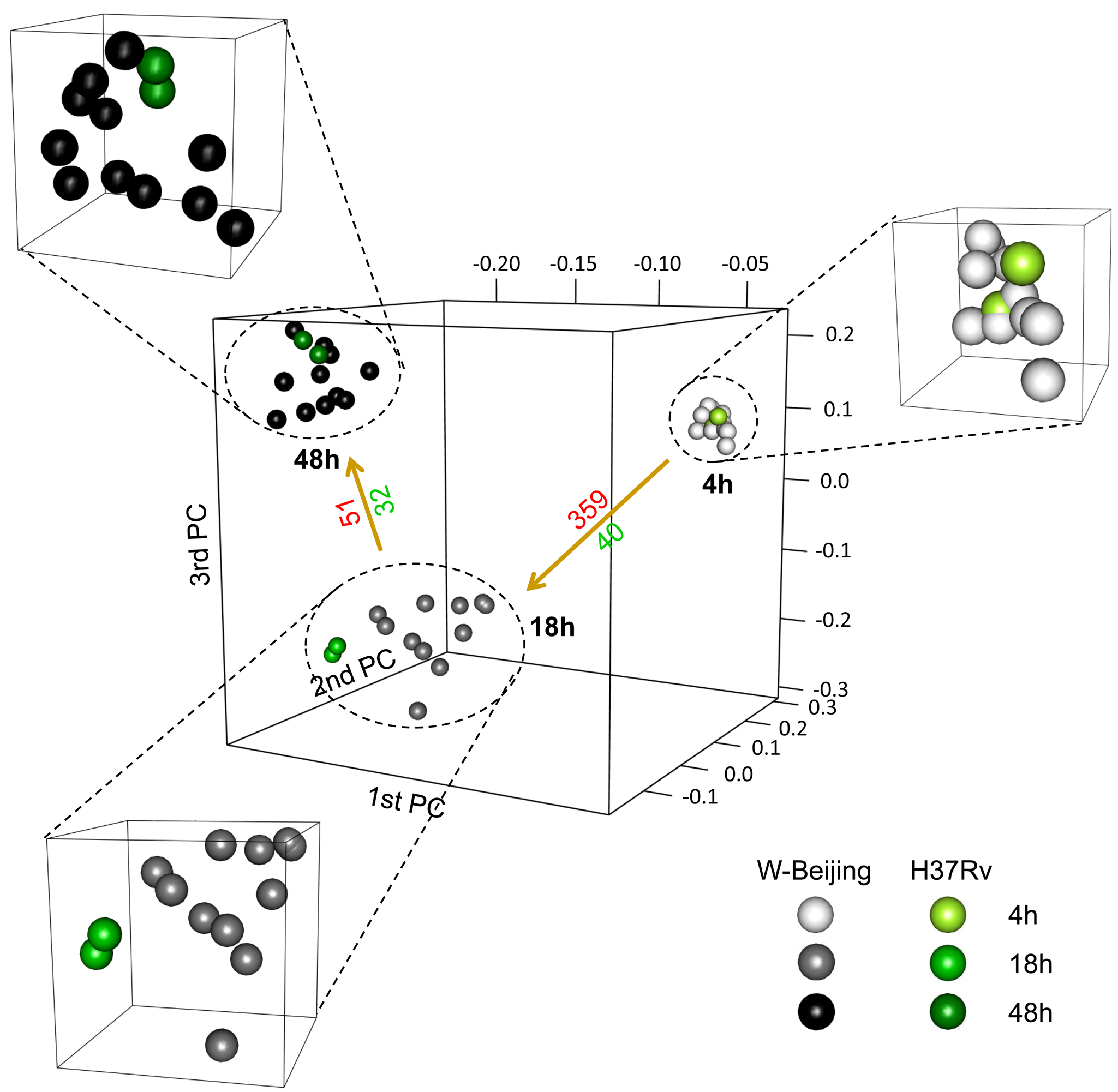

Supplement: Figure S1 — Principal component analysis of transcriptome of THP-1 cells infected by Mtb strains. Transcriptome profiles of THP-1 cells infected by W-Beijing strains as well as the lab strain H37Rv at different time points were colored as indicated. The number of those genes significantly upregulated and downregulated between any adjacent time points were colored red and green respectively, as identified using LIMMA. (TIF) [file pone.0038367.s001.tif]

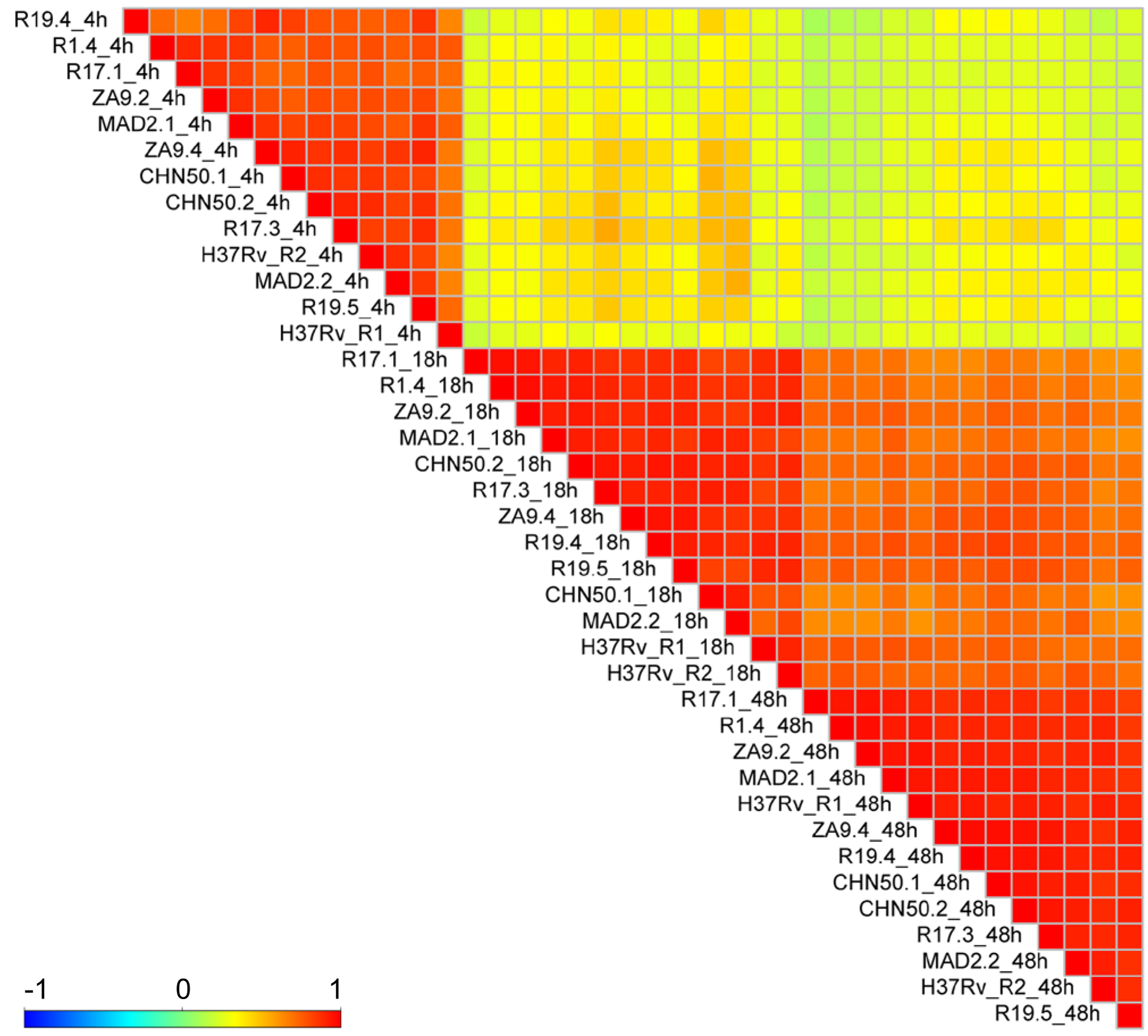

Supplement: Figure S2 — The visualization of pair-wise correlations between samples. The colors relate to Pearson’s correlation coefficient values, with deeper red colors indicating higher correlations. (TIF) [file pone.0038367.s002.tif]

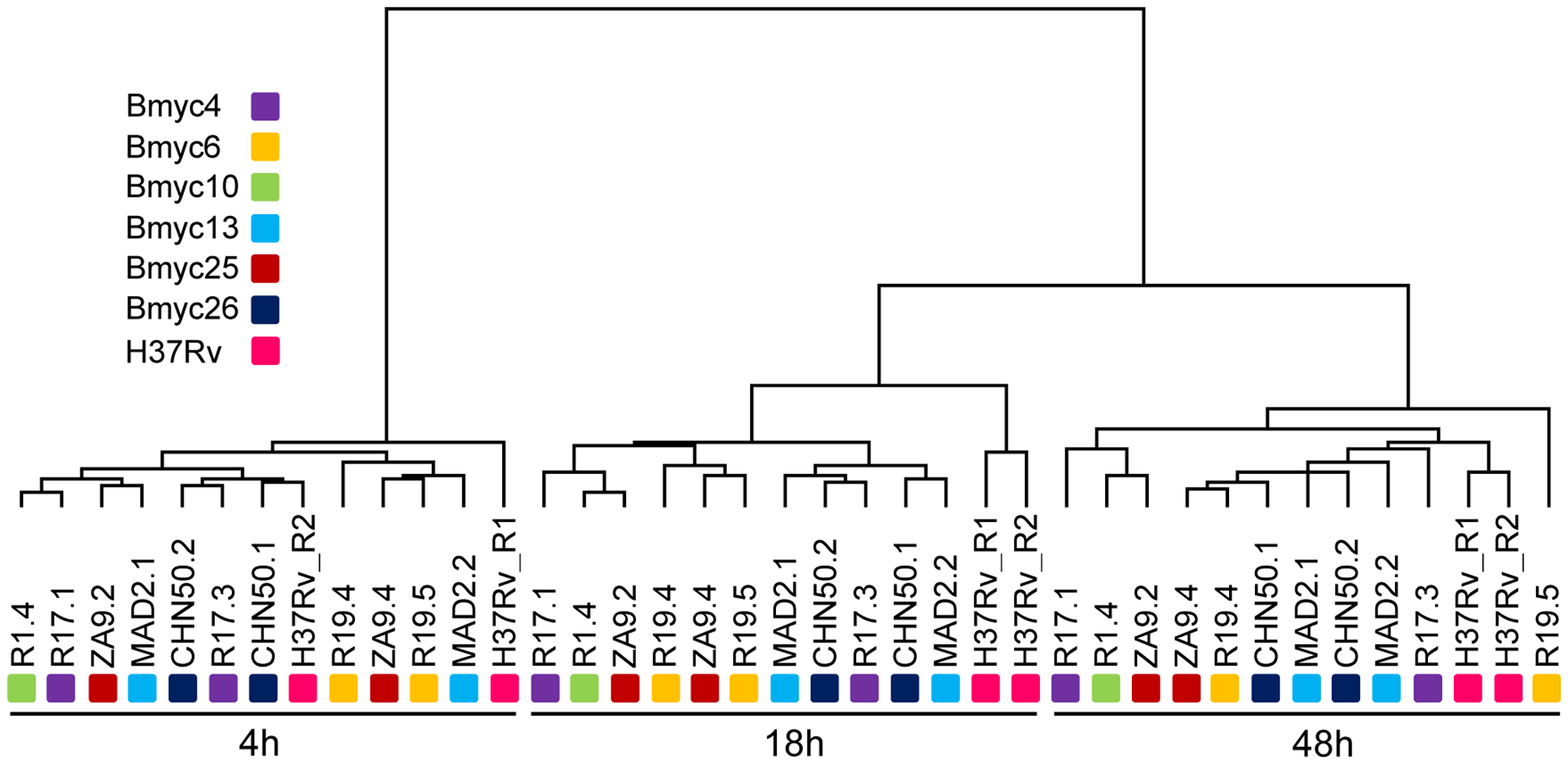

Supplement: Figure S3 — Sample classification based on genes annotated to immunity and defense functional categories. Immunity and defense related genes (1,091 probesets) was used for unsupervised sample classification. Samples infected by strains from the same node were indexed with the same color as in Figure 2A. (TIF) [file pone.0038367.s003.tif]

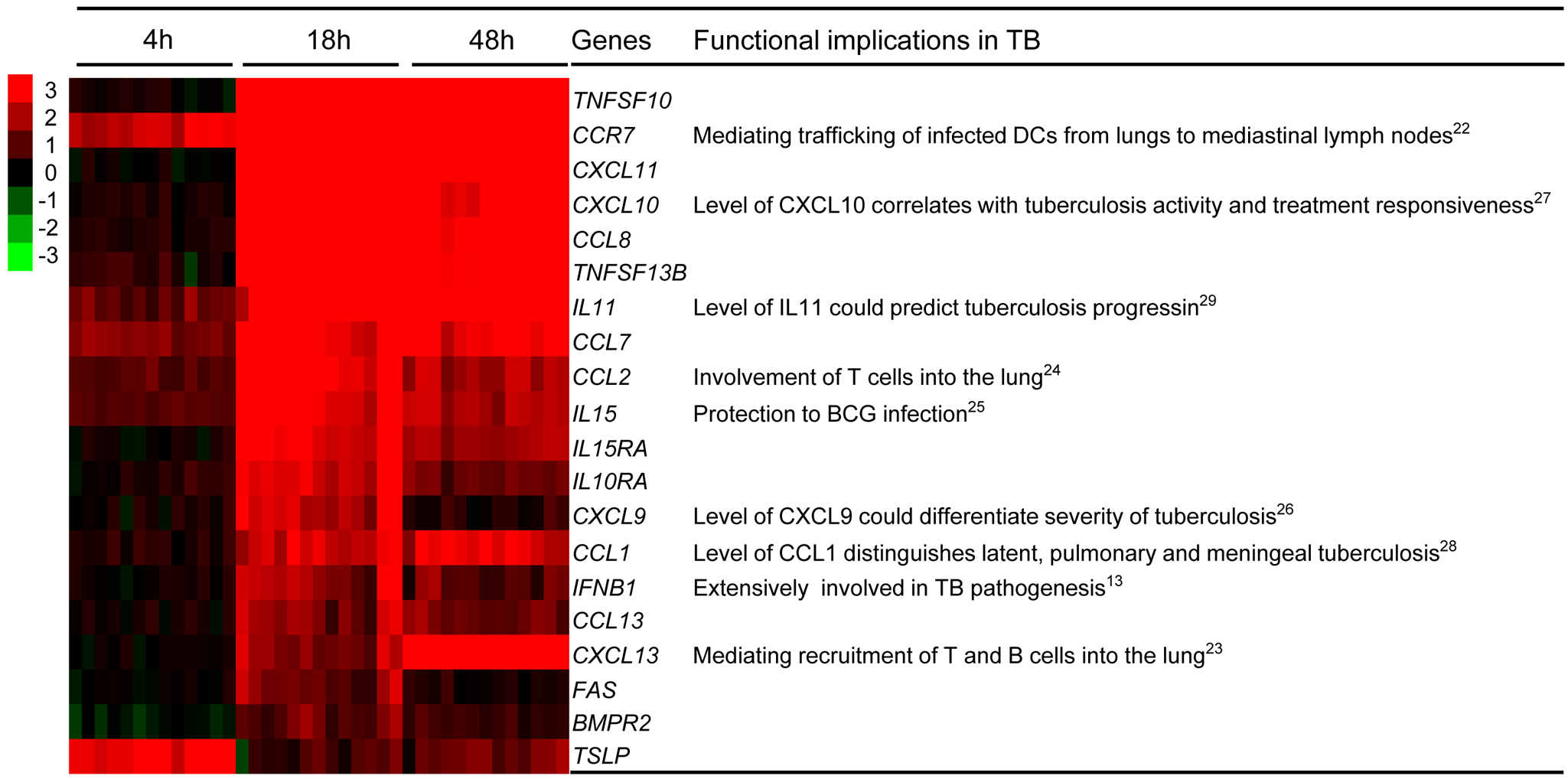

Supplement: Figure S4 — The functional implications of cytokine-cytokine receptor interactions in mediating host- Mtb cross-talk. Expression pattern (left panel) of genes involved in cytokine-cytokine receptor interaction and their functional implications in TB process (right panel). (TIF) [file pone.0038367.s004.tif]

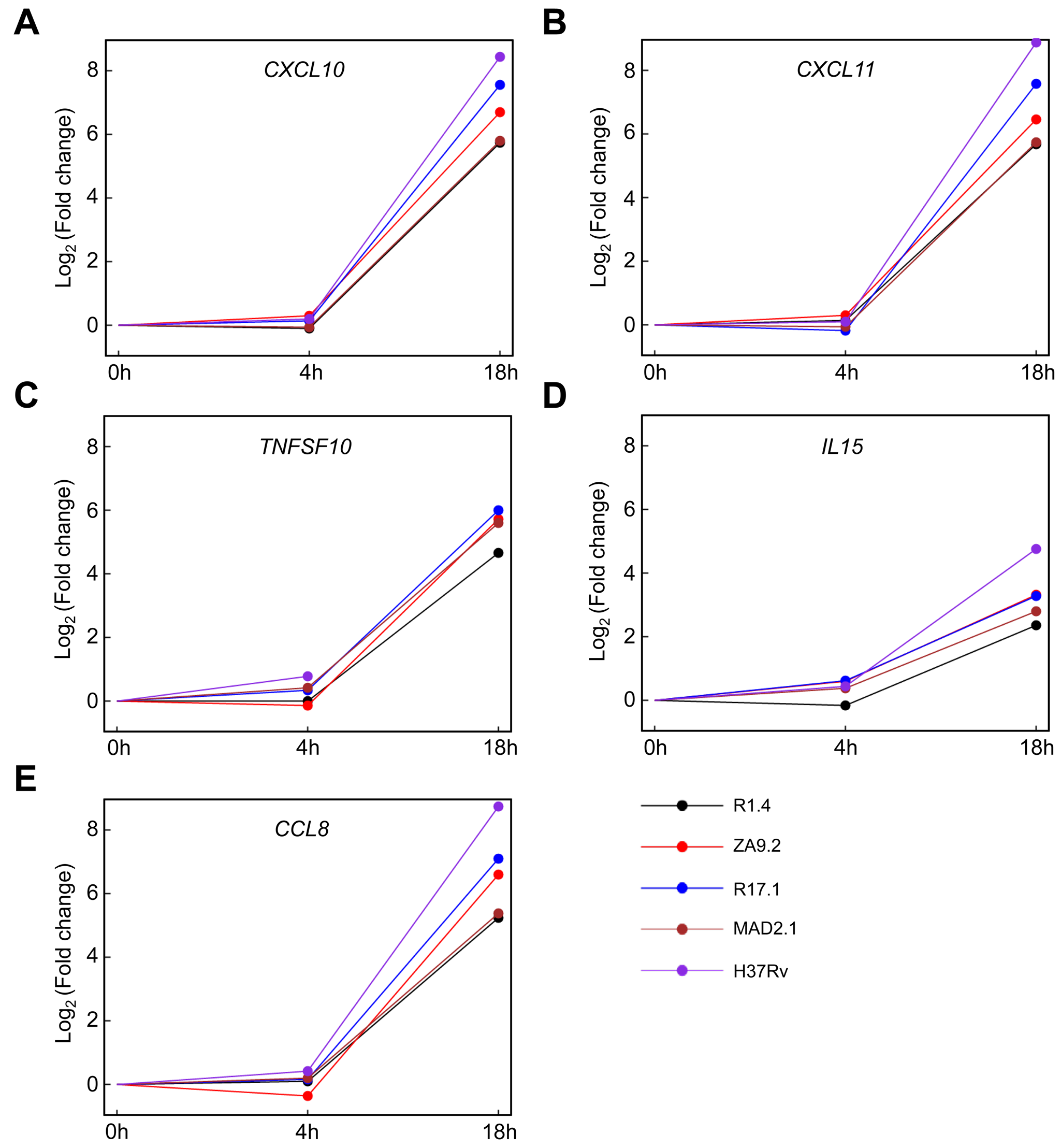

Supplement: Figure S5 — qRT-PCR validation of CXCL10 (A), CXCL11 (B), TNFSF10 (C), IL15 (D), and CCL8 (E). Expression of five genes were validated in five time-series samples infected by strains of R1.4, ZA9.2, R17.1, MAD2.1, and H37Rv. The fold changes at 4 h and 18 h of infection relative to 0 h (prior to infection) were log2-transformed. (TIF) [file pone.0038367.s005.tif]

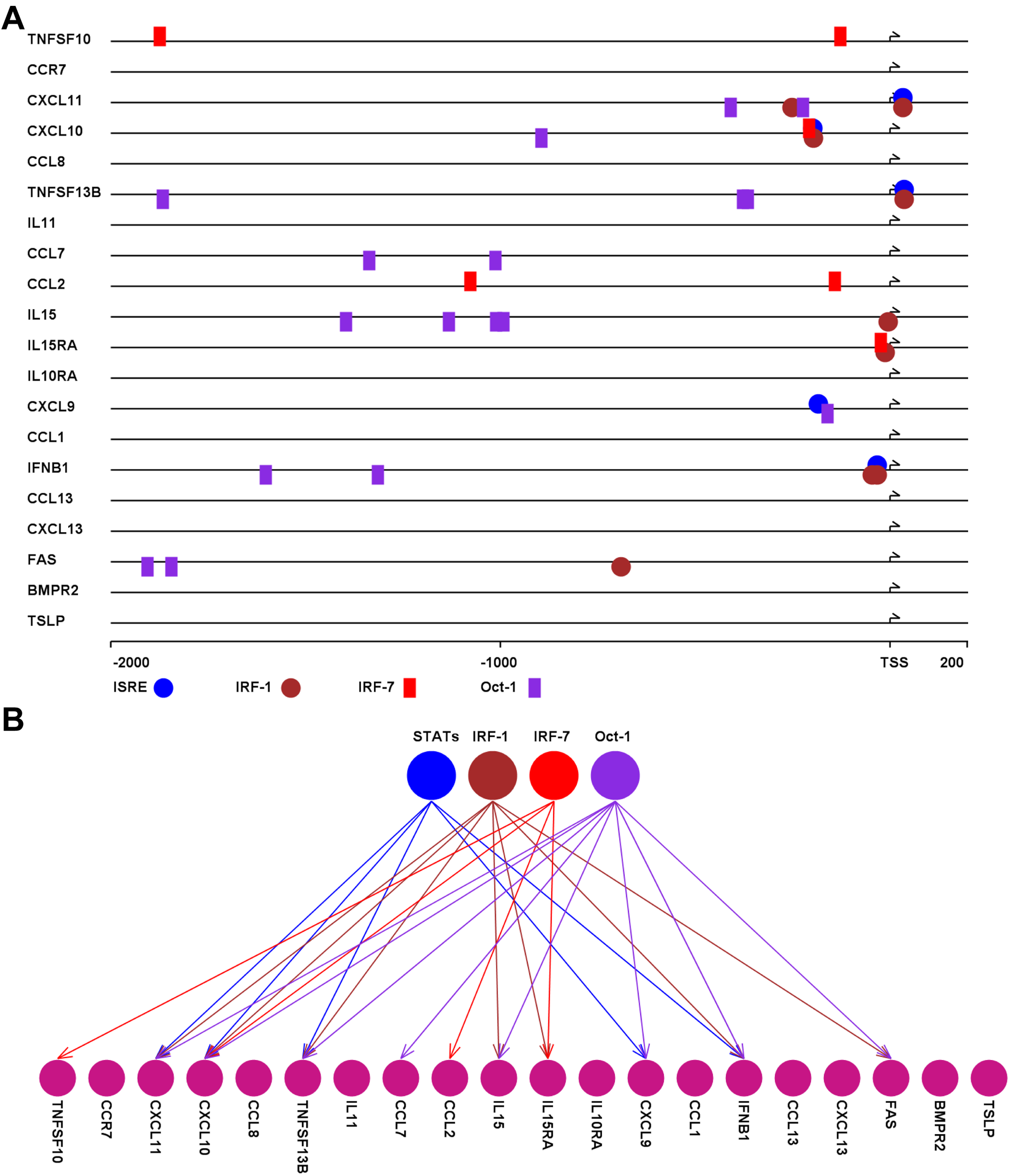

Supplement: Figure S6 — Potential involvement of STATs, IRF-1, IRF-7, and Oct-1 in cooperatively regulating cytokine-cytokine receptor interactions. (A) Relative locations of putative binding sites (ISRE, IRF-1, IRF-7, and Oct-1) in promoter regions of genes involved in cytokine-cytokine receptor interactions. (B) Putative target overlaps among STATs, IRF-1, IRF-7, and Oct-1 on genes in pathway of cytokine-cytokine receptor interactions. (TIF) [file pone.0038367.s006.tif]

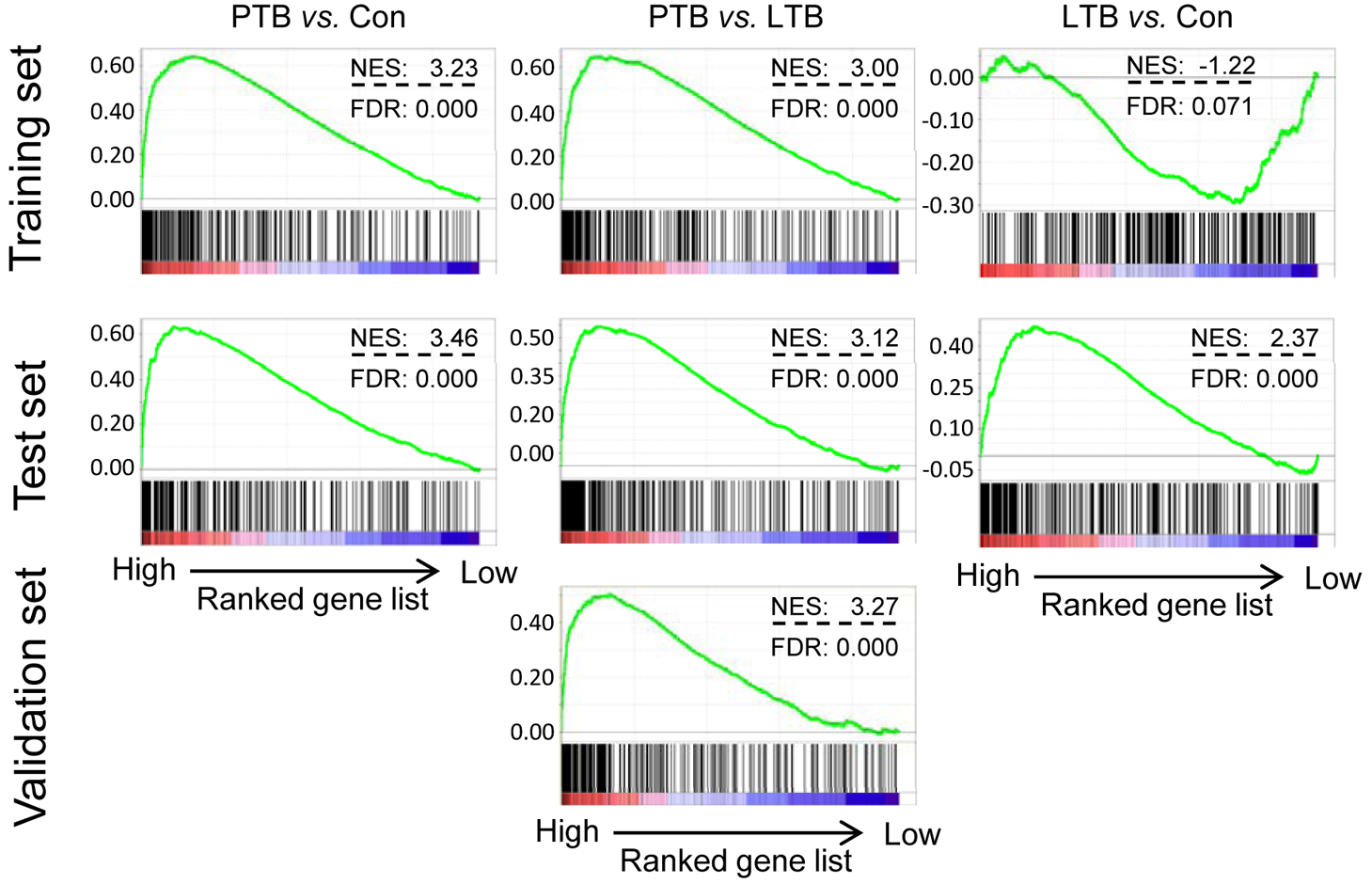

Supplement: Figure S7 — GSEA of the THP1r2 Mtb -induced signature using transcriptome data from tuberculosis patients with different disease degrees. (TIF) [file pone.0038367.s007.tif]

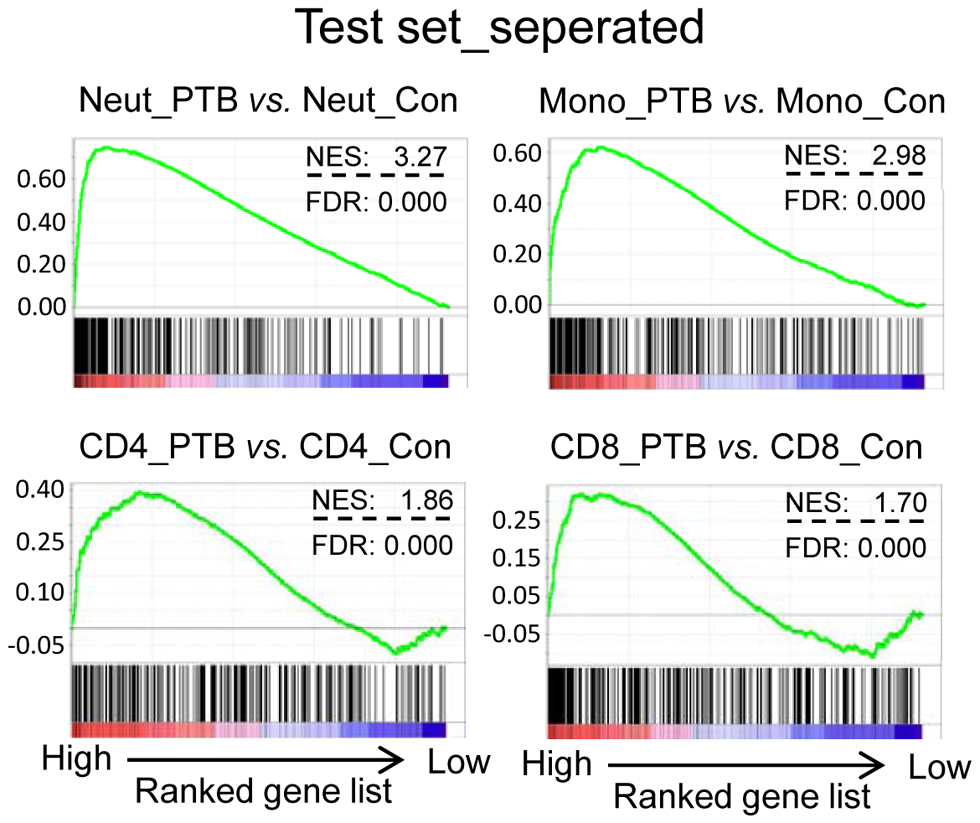

Supplement: Figure S8 — GSEA of the THP1r2 Mtb -induced signature using transcriptome data from different cell populations in PTB patients. (TIF) [file pone.0038367.s008.tif]

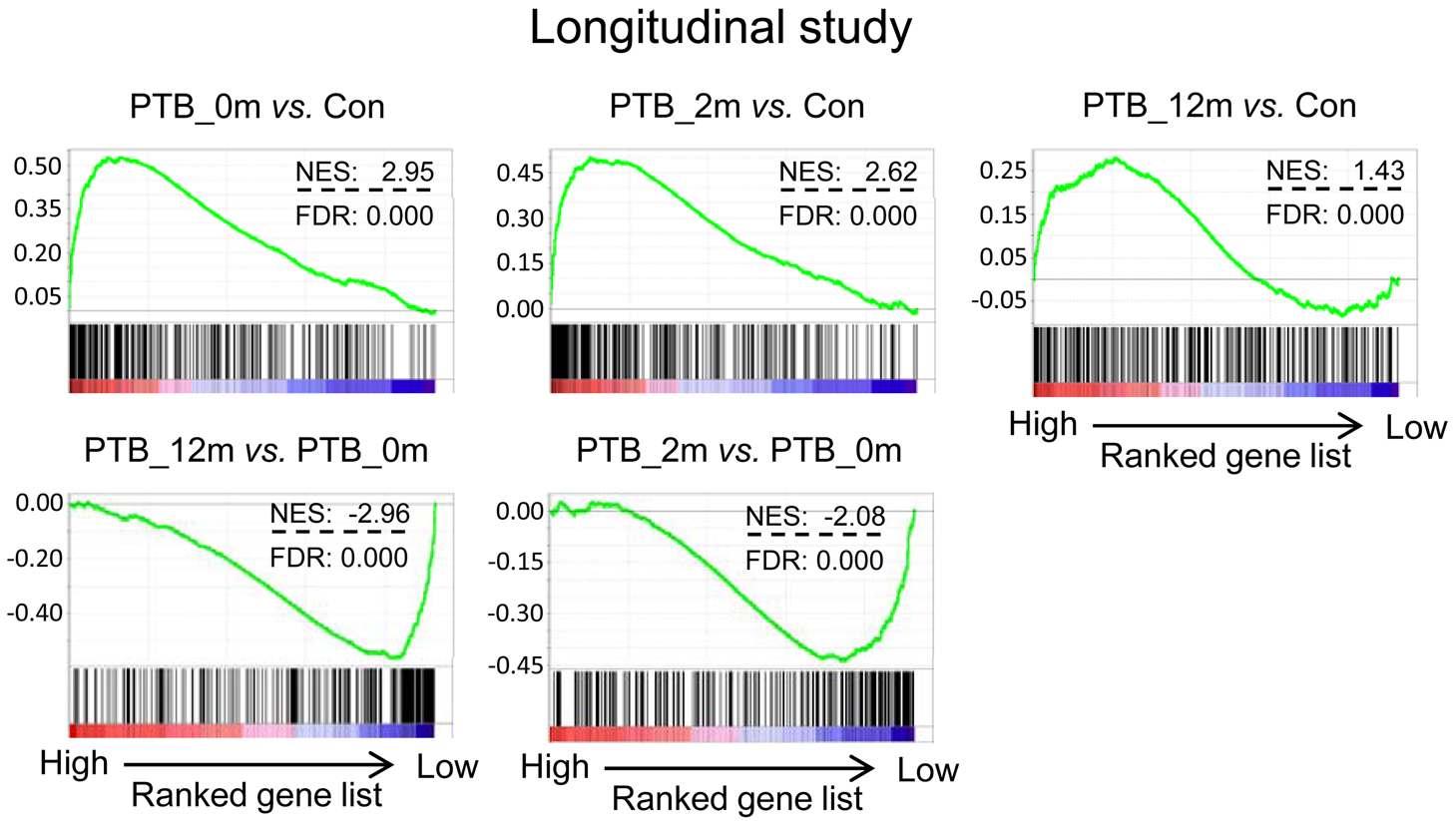

Supplement: Figure S9 — GSEA of the THP1r2 Mtb -induced signature using transcriptome data from tuberculosis patients with different therapy periods. (TIF) [file pone.0038367.s009.tif]

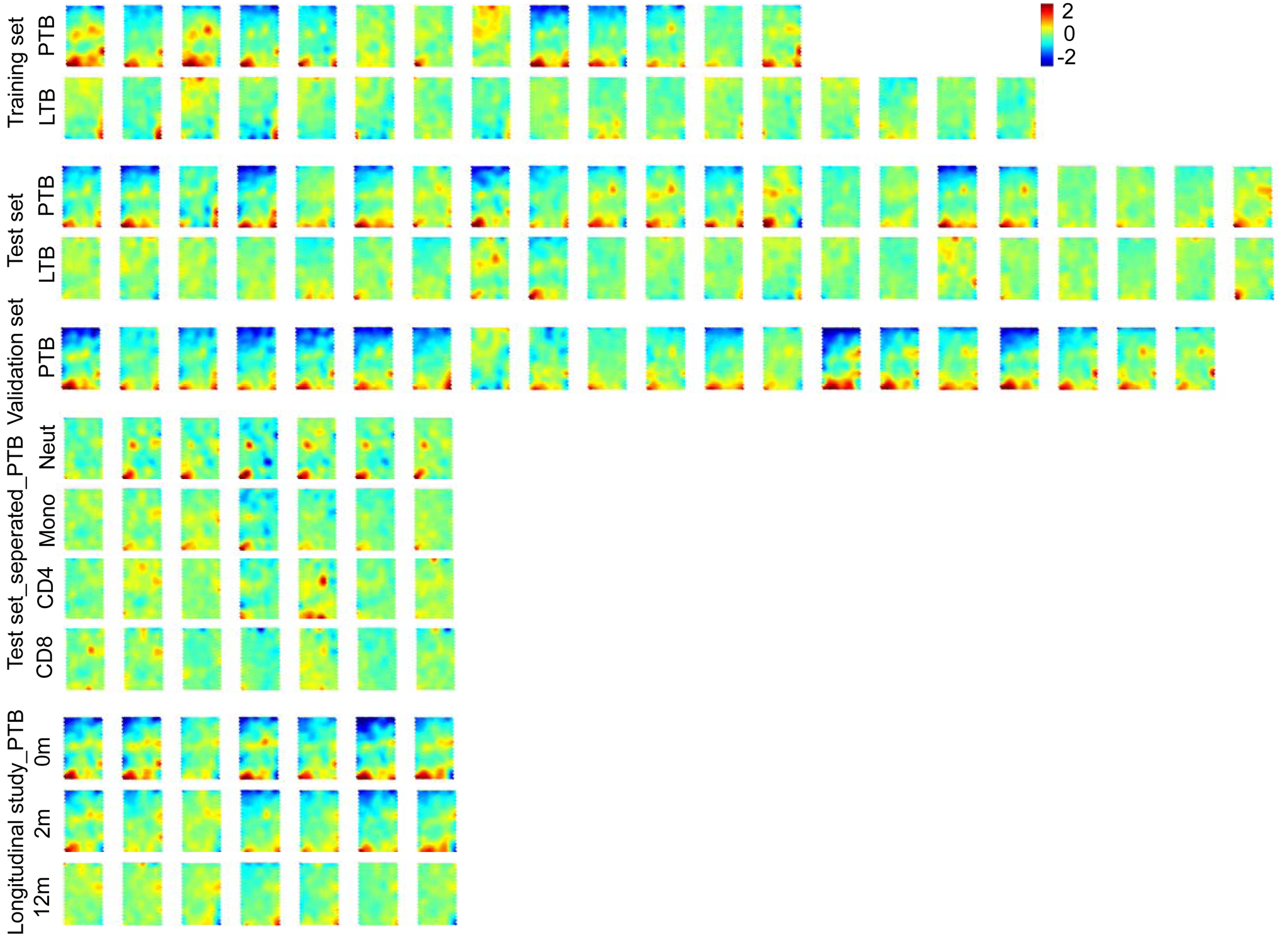

Supplement: Figure S10 — CPP-SOM of transcriptome data from human tuberculosis patients. The Series Matrix File, which contains expression data of 498 samples, was downloaded from NCBI GEO (accession number: GSE19491). The expression data from PTB, PTB with different therapy periods, LTB, or separated leucocyte populations of PTB were subtracted by their cognate healthy controls. Then, the subtracted profiles were visualized by CPP-SOM as in Figure 2B. (TIF) [file pone.0038367.s010.tif]

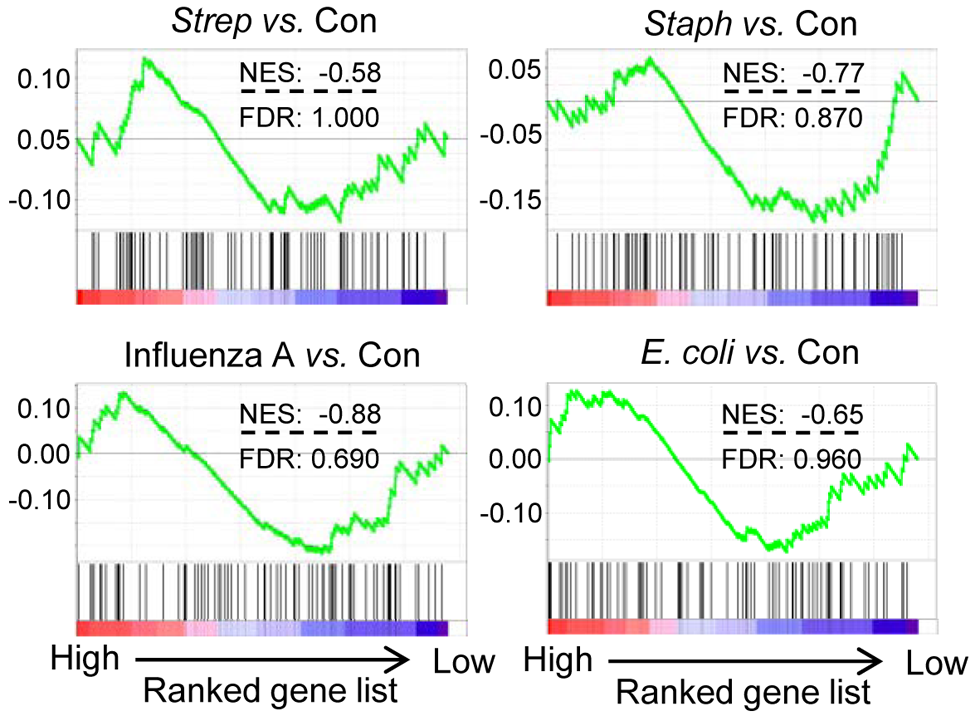

Supplement: Figure S11 — GSEA of the THP1r2 Mtb -induced signature using data from human patients with other acute infections. (TIF) [file pone.0038367.s011.tif]

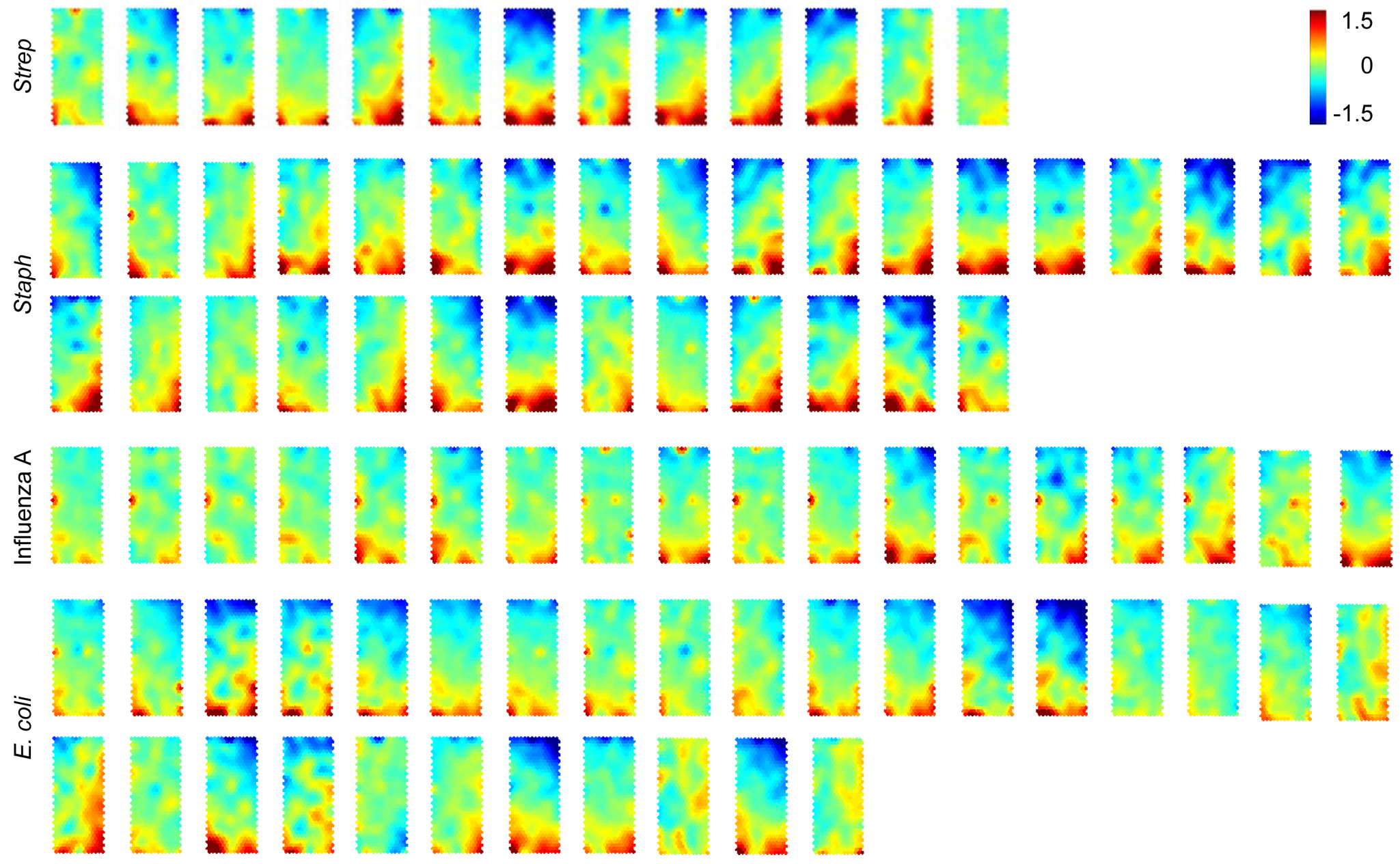

Supplement: Figure S12 — CPP-SOM of transcriptome data from human patients with other acute infections. The raw data were downloaded from NCBI GEO (accession number: GSE6269). The data were normalized and filtered in the same way as our THP-1 transcriptome data. The acute infection data were subtracted by the healthy controls, and visualized by CPP-SOM as in Figure 2B. (TIF) [file pone.0038367.s012.tif]

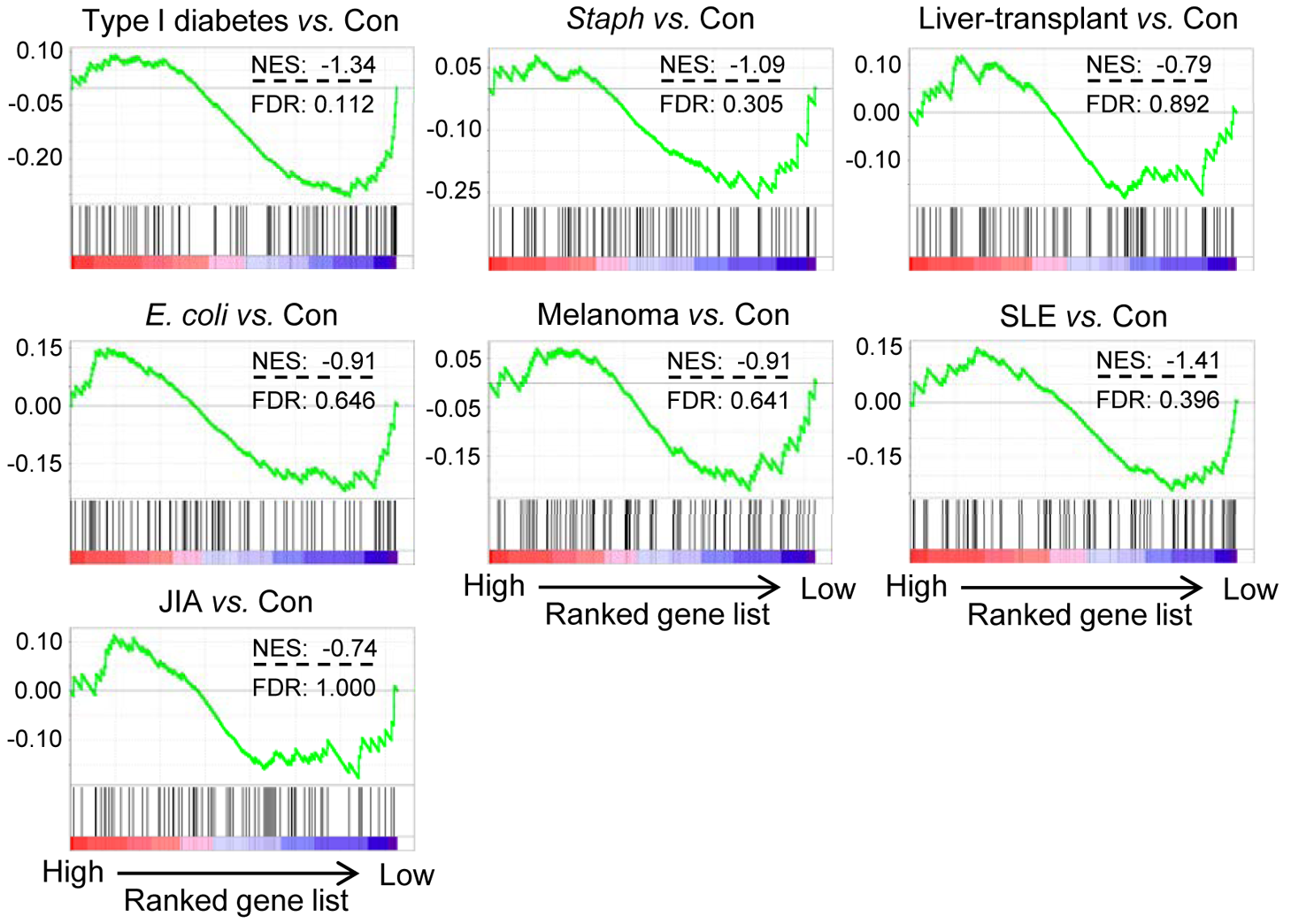

Supplement: Figure S13 — GSEA of the THP1r2 Mtb -induced signature using transcriptomes from patients with other inflammatory or pathological conditions. (TIF) [file pone.0038367.s013.tif]

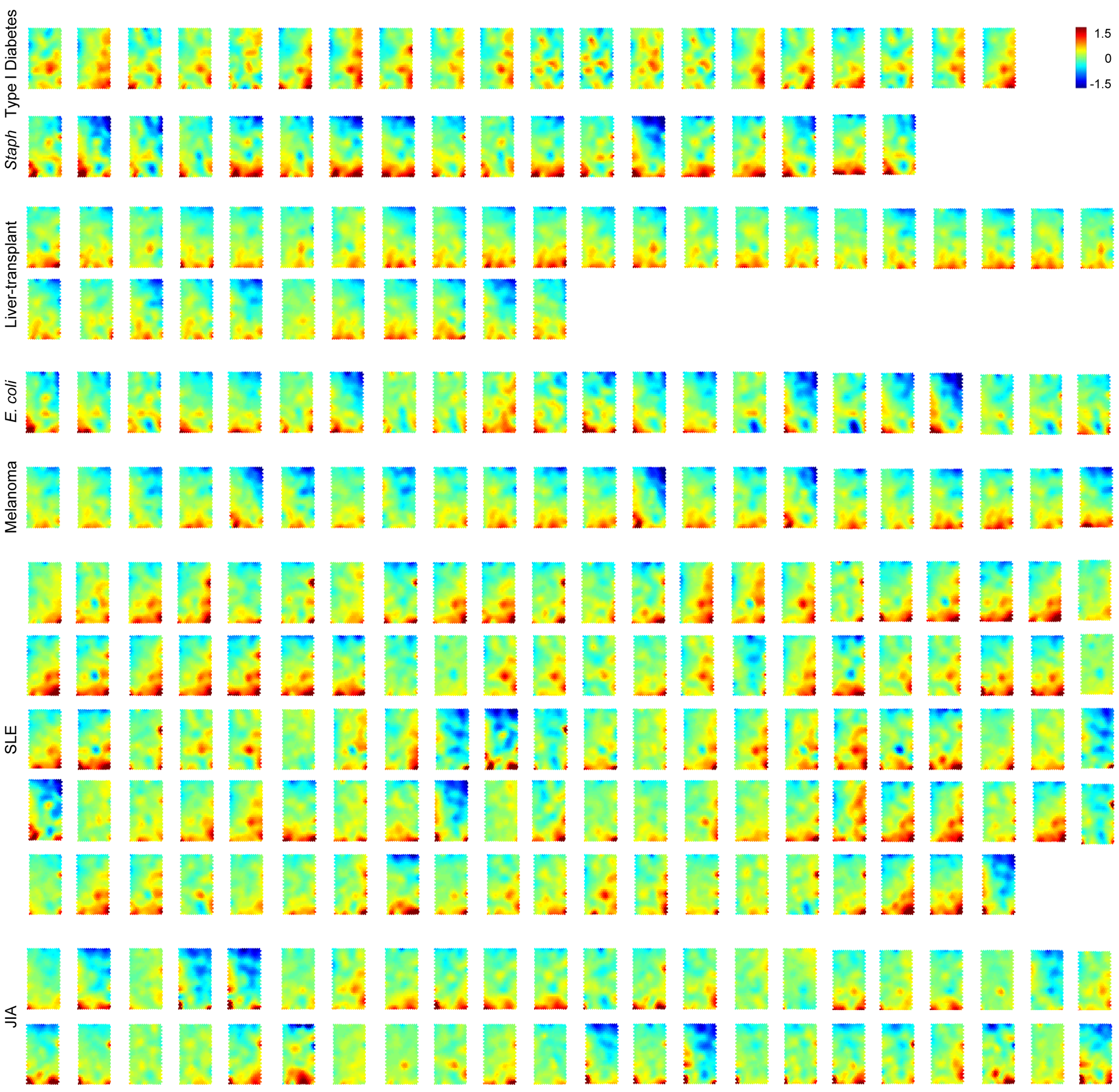

Supplement: Figure S14 — CPP-SOM of transcriptome data from patients with other inflammatory or pathological conditions. The raw data were downloaded from NCBI GEO (accession number: GSE11907). The data were normalized, filtered using the same as our THP-1 transcriptome data. (TIF) [file pone.0038367.s014.tif]
